# Semaglutide-associated risk of nonarteritic anterior ischemic optic neuropathy in patients with type 2 diabetes: A systematic review and meta-analysis of observational studies

**DOI:** 10.1371/journal.pmed.1005064

**Published:** 2026-05-21

**Authors:** Jędrzej Chrzanowski, Magdalena Walicka, Jacek Burzyński, Małgorzata Zaraś, Arkadiusz Michalak, Wojciech Fendler

**Affiliations:** 1 Department of Biostatistics and Translational Medicine, Medical University of Lodz, Lodz, Poland; 2 Department of Internal Diseases, Endocrinology and Diabetology, National Medical Institute of the Ministry of the Interior and Administration, Warsaw, Poland; 3 Department of Human Epigenetics, Mossakowski Medical Research Institute, Polish Academy of Sciences, Warsaw, Poland; 4 Ophthalmology Department, CMP Medical Center, Warsaw, Poland; 5 Department of Pediatrics, Diabetology, Endocrinology and Nephrology, Medical University of Lodz, Lodz, Poland; Wuqu’ Kawoq | Maya Health Alliance, GUATEMALA

## Abstract

**Background:**

Semaglutide, a glucagon-like peptide-1 receptor agonist, is widely used for the management of type 2 diabetes (T2DM). Recent case reports have raised concerns about a potential association between semaglutide use and the development of nonarteritic anterior ischemic optic neuropathy (NAION), a rare but vision-threatening condition. We aimed to evaluate whether semaglutide use is associated with an increased risk of NAION in patients with T2DM.

**Methods and findings:**

We conducted a systematic review and meta-analysis of observational studies comparing patients with T2DM aged ≥12 years treated with semaglutide to those receiving other glucose-lowering therapies. We searched PubMed, Scopus, and Web of Science databases from January 2023 to November 2025. Two reviewers independently extracted data on study design, population characteristics, and outcomes. Risk of bias was assessed using the Newcastle–Ottawa Scale, and ROBINS-I v.2. Certainty of the evidence was graded according to the GRADE framework. Pooled hazard ratios (HRs) and 95% confidence intervals (CIs) were calculated using fixed-effects models; sensitivity analyses included crude and subgroup HRs, and overlapping study replacement. Leave-one-out analysis was conducted to assess small-study effects and publication bias. Results were contextualized within other meta-analyses, systematic reviews, consensus statements, and regulatory communications on the topic.

Five eligible observational studies met the inclusion criteria, and 7 additional studies were included in the sensitivity analysis. Semaglutide use was associated with a significantly increased hazard of NAION compared with nonsemaglutide glucose-lowering regimens (HR 2.17, 95% CI [1.73, 2.74]; *p* < 0.001), regimens excluding other GLP-1 receptor agonists (HR 2.13, 95% CI [1.60, 2.83]; *p* < 0.001), and sodium-glucose co-transporter 2 inhibitor (SGLT2i) users (HR 1.96, 95% CI [1.28, 2.99]; *p* = 0.002). Despite the increased relative risk, the absolute risk remained low at 0.014% (95% CI [0.005%, 0.023%]; *p* = 0.002), corresponding to approximately 1 additional case of NAION per 7,000 semaglutide-treated patients annually. The results were consistent with meta-analyses of observational studies and corroborated decisions presented in regulatory communications. Due to exclusive focus on retrospective, registry-based observational studies, the evidence synthesis was limited and could be biased by study-level outcome misclassification and confounding.

**Conclusions:**

Our findings suggest a possible association between semaglutide use and an increased risk of NAION in patients with T2DM. Although the absolute risk is low, clinicians should be aware of this potential adverse event, particularly in individuals at increased baseline risk for optic neuropathies. While these findings support current recommendations to discontinue semaglutide in patients diagnosed with NAION, the certainty of the available evidence is low, underscoring the need for further high-quality studies to clarify this association.

## Author summary

**Why was this study done?:**

- Semaglutide, a glucagon-like peptide-1 receptor agonist, is established in the management of type 2 diabetes and has more recently emerged as a prominent therapeutic option for obesity.
- As semaglutide’s cumulative exposure grows, studies report on its new potential very rare adverse reaction–nonarteritic anterior ischemic optic neuropathy (NAION), which is a rare but vision-threatening condition.
- Current, mostly registry and pharmacovigilance evidence, along with recent meta-analyses, indicate a possible link of semaglutide with NAION incidence, though the exact association remains not fully elucidated. Synthesis with a specific focus on bias and confounding management is necessary.

**What did the researchers do and find?:**

- We performed the meta-analysis of longitudinal observational real-world studies reporting time-to-event outcomes (5 primary studies, 7 additional studies for sensitivity analysis) to determine whether semaglutide therapy is associated with an increased risk of NAION in patients with T2D. We also compared our data with other meta-analyses (2 for randomized controlled trials [RCT], 4 for observational studies), systematic reviews (9), consensus statements (2), and regulatory communications (4).
- Semaglutide treatment significantly increased the hazard for NAION compared to other antidiabetic treatments, translating to 1 additional NAION case per 7000 patients treated for 1 year.
- Our results were in line with other observational meta-analyses, while meta-analyses of RCTs consistently reported fewer NAION events.

**What do these findings mean?:**

- The pooled HR estimates from our meta-analysis support the conclusions of the European Medicines Agency and the World Health Organization, which classified NAION as a “very rare” (up to 1 in 10,000 users per year) side effect of semaglutide.
- In our meta-analysis, we included studies that relied on retrospective, registry-based designs with International Classification of Diseases codes to identify NAION cases, introducing potential misclassification and confounding by indication. As the included studies were assessed as having a moderate to serious risk of bias and the overall certainty of evidence was graded as low to very low, these findings should be interpreted with caution. More high-quality, prospective studies employing standardized NAION diagnostic criteria are needed to confirm these results and to better identify and protect individuals at the greatest risk.

## Abstract 3

In a systematic review and meta-analysis of observational studies, Jędrzej Chrzanowski and colleagues investigate whether there is an association between semaglutide use and the development of non-arteritic anterior ischemic optic neuropathy (NAION).

## Introduction

Semaglutide is a potent glucose-lowering agent that functions as a glucagon-like peptide-1 receptor agonist (GLP-1 RAs). Although initially developed for the management of type 2 diabetes [1], subsequent studies have demonstrated that the therapeutic benefits of GLP-1 RAs extend beyond glycemic control [2,3], with higher doses associated with clinically meaningful efficacy in the treatment of obesity. Its use is supported by robust evidence showing substantial reductions in cardiovascular risk [4–9], and semaglutide is now recommended in many international guidelines [10–13]. As its indications expand and cumulative exposure grows [14], careful evaluation of rare but potentially serious adverse events becomes increasingly important [2].

Recently, several registry-based cohort studies [15–28], pharmacovigilance reports [29–33], and case series [34–40] have reported an association between semaglutide therapy and nonarteritic anterior ischemic optic neuropathy (NAION)—a rare condition with an incidence of approximately 3.89–10.19 cases per 100,000 patient-years in the general population [41], characterized by sudden, painless unilateral vision loss due to acute ischemia of the anterior portion of the optic nerve [42]. Patients with type 2 diabetes treated with semaglutide were found to be at an elevated risk for developing NAION, with adjusted hazard ratios (HRs) ranging from ~1.26 to 6.84, and increased incidence, particularly in the first 12 months after treatment initiation [15–26].

The causal link between semaglutide treatment and NAION remains uncertain. Many established NAION risk factors—older age, hypertension, hyperlipidemia, diabetes mellitus, coronary heart disease, obstructive sleep apnea, and smoking—mirror indications for semaglutide use, raising concern about confounding by indication and disease severity [43–51]. Furthermore, meta-analyses of randomized controlled trials (RCTs) of GLP-1 RAs have not demonstrated a statistically significant increase in NAION [52,53]. However, event counts are extremely small (due to low absolute risk of NAION), and trial populations may under-represent some of the highest-risk individuals routinely treated in practice.

Amid this uncertainty, regulators have recently moved from treating NAION as a signal under evaluation to a labelled, very rare adverse reaction of semaglutide [54–60]. The European Medicines Agency’s (EMA) Pharmacovigilance Risk Assessment Committee concluded that semaglutide approximately doubles the risk of NAION, corresponding to about 1 excess case per 10,000 person-years of exposure, and recommended discontinuing treatment if NAION is diagnosed. The World Health Organization (WHO) has issued a coordinated global safety communication, advising urgent ophthalmic assessment and discontinuation of semaglutide if patients experience sudden vision loss.

Considering this evolving and sometimes conflicting evidence, we conducted a systematic review and meta-analysis of longitudinal observational studies to quantify the association between semaglutide use and NAION in patients with type 2 diabetes. We provide a comprehensive evaluation of all eligible studies (S1–S3 Tables) and perform a synthesis of evidence, taking into consideration both the choice of comparators (any other treatment; specifically excluding other GLP-1 RAs; specifically, sodium-glucose cotransporter 2 inhibitors [SGLT2i]) and overlap in populations across studies (S5 Table). We have also included a comprehensive overview of other available systematic reviews and meta-analyses on the topic as an umbrella review (S6–S9 Tables).

## Methods

### Primary systematic review and meta-analysis

The primary objective of this systematic review and meta-analysis was to determine whether semaglutide therapy is associated with an increased risk of NAION in patients with type 2 diabetes compared with other antidiabetic treatment regimens, based on data from longitudinal observational real-world studies that report time-to-event outcomes. To evaluate this impact, we conducted a systematic review in accordance with the Methodological Expectations of Cochrane Intervention Reviews (MECIR) standards [61], and reported the results in accordance with the PRISMA 2020 guidelines [62]. The review was registered in the International Prospective Register of Systematic Reviews (PROSPERO) no. CRD420251025896 with further amendments.

A comprehensive literature search was conducted in PubMed, Scopus, and Web of Science databases for studies published between January 2023 and April 2025, with a repeat search after the first round of manuscript review, including studies published through November 2025. The search strategy combined Medical Subject Headings and keywords related to semaglutide, GLP-1 receptor agonists, and NAION. The search terms are reported in the PROSPERO protocol (S1 PROSPERO Protocol). Additional relevant articles were identified through manual screening of reference lists and citation tracking of key publications. Only studies published in English were included; however, the sensitivity search identified no other relevant manuscripts published in other languages [63–65].

In this systematic review and meta-analysis, we focused exclusively on longitudinal observational studies that evaluated the risk of NAION associated with semaglutide use in patients aged 12 years or older with type 2 diabetes, including prospective, retrospective, and registry-based studies.

Exclusion criteria were RCTs, other study designs, nonpeer-reviewed publications, and studies without clear documentation of semaglutide exposure or other lack of NAION-specific outcome. The exclusion of RCTs was to focus specifically on the early reports of the NAION-semaglutide link, and to describe and quantify the potential bias. For registry-based studies, to avoid including studies with overlapping populations, we retained the largest, most comprehensive cohort to avoid double-counting (S5 Table) and substituted it with equivalent population studies as a sensitivity analysis.

Due to the complexity and incomplete reporting of multidrug regimens used in the treatment of type 2 diabetes, it was difficult to define specific comparator groups. As such, comparator groups were defined as hierarchical treatment groupings: (A) treatment other than semaglutide (could include nonsemaglutide GLP-1 RAs); (B) other than GLP-1 RAs; and (C) specifically SGLT2i treatment. The primary outcome was the hazard ratio for NAION. The outcome definition was primarily based on International Classification of Diseases (ICD-10) diagnostic codes, with some studies verifying NAION against source medical documentation [15]. Studies identified semaglutide treatment using national prescription registries, though Cai and colleagues [19] and Hathaway and colleagues [15] did not provide detailed information on the definitions of semaglutide use. To minimize dependence, we included at most one effect size per study within each comparator stratum. Any residual dependence was acknowledged as a limitation. For details, see S2 Table. Duplicates were removed. Two reviewers (JC, MW) independently screened titles and abstracts—studies that did not meet the inclusion criteria were excluded at this stage. Full-text articles were retrieved and assessed by the same reviewers. Disagreements were resolved by discussion with the arbiter (JB). Reasons for exclusion of full-text articles were documented in the S3–S5 Tables. Data were extracted using a standardized form by two independent reviewers (JC, MW). Extracted information included study characteristics (author, year, design, setting, and sample size), participant demographics (age, sex, and diagnosis), intervention and comparator details, measures for developing NAION (adjusted HR with 95% confidence interval [95%CI] for semaglutide use against comparator, incidence for treatment groups if available—for details on confounding variables and additional adjustment methods see S2 Table), and follow-up duration. In cases with multiple NAION episodes, only the time to the first event was included in subsequent analyses. No missing data handling or data-conversion methods were applied. Risk of bias was assessed using the Newcastle-Ottawa Scale (NOS) [66] and Risk of Bias in Non-Randomized Studies of Interventions (ROBINS-I, version 2) [67]. Assessments were performed independently by two reviewers (JC, MW), with disagreements resolved by a third reviewer (JB).

Results were synthesized using Cochrane’s Review Manager (RevMan, v9.5.1, Cochrane Collaboration) software and using R (v.4.5.1) with metafor package (v.4.8). Code for reproducibility is available in Zenodo (https://doi.org/10.5281/zenodo.17885011).

Primary analysis was conducted using inverse-variance fixed-effects (Wald CI), with sensitivity analyses using the Hartung–Knapp random-effects method [68]. Heterogeneity was assessed with Cochran’s Q test and reported as I² with 95% CI and *p*-value [69,70]. Sensitivity analyses also included alternative synthesis scenarios using unadjusted HR, other NAION definition criteria, and consideration of replacement studies (defined as samples from the overlapping source population). Small study effects and reporting bias were assessed using leave-one-out sensitivity testing [71]. The certainty of the evidence was evaluated using the Grading of Recommendations Assessment, Development and Evaluation (GRADE) [72] as implemented in GRADEpro GDT by two reviewers (JC, MW), with disagreements resolved through consensus.

### Umbrella review

To complement the primary meta-analysis, we conducted an umbrella review of systematic reviews, structured narrative reviews, meta-analyses, expert consensus statements, and regulatory safety communications on the link between semaglutide or other GLP-1 RAs and NAION. We followed the JBI Manual for Evidence Synthesis [73] and Preferred Reporting Items for Systematic reviews and Meta-Analyses (PRISMA 2020) [62]. A literature search was conducted in parallel with the primary meta-analysis, using the same databases (PubMed, Scopus, and Web of Science) and time frame (January 2023–November 2025). Additional sources (WHO, EMA, and Food and Drug Administration [FDA]) were identified through targeted grey literature searches and citation tracking. Two reviewers (JC, JB) independently screened and retrieved full texts. Eligible publications were required to (i) use systematic methods for study identification, selection, or appraisal; and (ii) examine ocular safety outcomes relevant to NAION in the context of semaglutide, GLP-1 RAs, or related agents. Commentaries and nonmethodological narrative summaries were excluded unless issued by regulatory agencies or professional societies.

Data were extracted using a structured form including review type, objective, population, intervention, comparators, included study designs, synthesis methods, and principal findings. Methodological quality of each review was appraised using A Measurement Tool to Assess Systematic Reviews (AMSTAR 2 [74]) by two reviewers (JC, JB), with discrepancies resolved by consensus.

Because of heterogeneity in scope, analytical approaches, and outcome definitions, no quantitative pooling across reviews was performed. Findings were synthesized narratively and grouped by evidence type (S6–S9 Tables). The umbrella review results were used to contextualize the primary meta-analytic estimates, assess consistency of effect direction across evidence tiers, and evaluate the extent of concordance with regulatory assessments.

## Results

### Study selection

**Fig 1:**
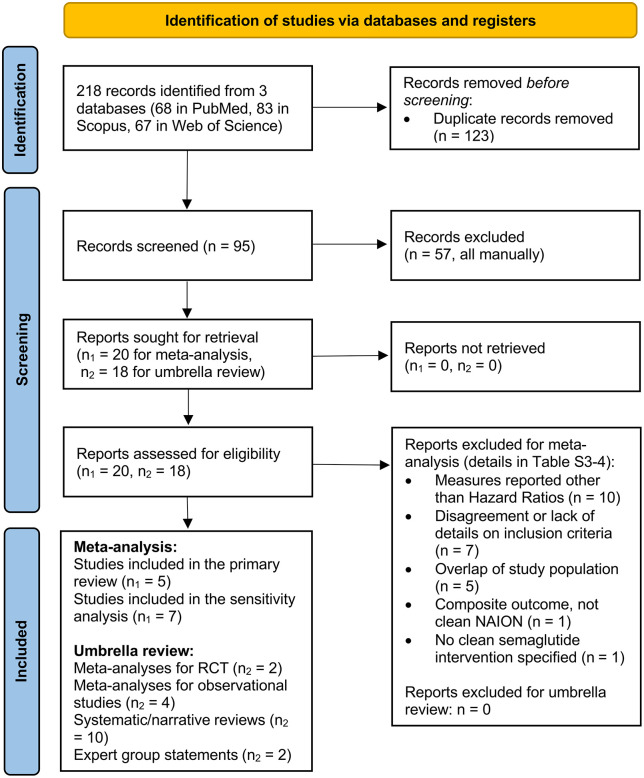
PRISMA flow diagram.

The study selection process is summarized in Fig 1. Our search strategy identified 218 records across 3 databases. After removing duplicates, 95 records were screened based on titles and abstracts, with a brief review of the full text, resulting in the exclusion of 57 records. The remaining 38 full-text reports (20 for meta-analysis, 18 for umbrella review) were assessed in depth for eligibility.

### Meta-analysis *of* observational studies

For meta-analysis, we selected only studies that reported time-to-event measures. As such, 15 studies were excluded, based on inappropriate event reporting (10), inclusion criteria (7), improper intervention (1), composite outcome (1), or overlapping populations (5)—for details see S3, S4 Tables. The remaining 5 studies were included in the meta-analysis, whereas the 7 studies excluded from the primary analysis were additionally used in the sensitivity analyses (S3 Table). Key characteristics of included studies are summarized in S1 Table.

The majority of included studies were registry-based and enrolled patients aged >18 years. Two studies additionally included younger patients (≥12 years old, Table 1) [15,16], and one considered in the sensitivity analysis included only older adults (>65 years old) [21]. Only two studies applied extra criteria for NAION classification beyond ICD-10 codes (H47.0 and H47.0c). Most studies provided a 5-year follow-up [16–18], while the other 2 or 3 years [15,19]. Simonsen and colleagues [18] reported data from two separate registries in Denmark and Norway; therefore, the cohorts were included as separate entries, with particular attention to avoid overlap with the cohort described by Grauslund and colleagues [17].

**Table 1:** Characteristics of selected studies.

Study	Design	Population	Intervention	Comparator	Outcome	Follow-up	NAION incidence (intervention group)
**Cai, 2025 [19]**	Registry	>18-year-old, type 2 diabetes	Semaglutide (*N* = 43,620)	A) Dulaglutide (*N* = 14,923) B) Glipizide (*N* = 44,092) C) Sitagliptin (*N* = 19,581) D) Empagliflozin (*N* = 43,302)	NAION (ICD10 codes twice in 90-days)	~2 years	14.5 per 100,000 py
**Hsu, 2025 [16]**	Registry	≥12-year-old, type 2 diabetes	Semaglutide (*N* = 174,584)	non-GLP-1 RAs (*N* = 174,584)	NAION (ICD10)	~5 years	0.026%
**Simonsen, 2025 [18]**	Registry	>18-year-old, type 2 diabetes	Semaglutide (*N* = 44,517, 16,860)	SGLT2i (*N* = 84,814, 34,153)	NAION (ICD10: H47.0, H47.0c)	~5 years	Denmark: 21.9 per 100,000 py Norway: 29.0 per 100,000 py
**Grauslund, 2024 [17]**	Registry	>18-year-old, type 2 diabetes	Semaglutide (*N* = 106,454)	Any other treatment (*N* = 317,698)	NAION (ICD10: H47.0c)	~5 years	22.8 per 100,000 py
**Hathaway, 2024 [15]**	Case-control	≥12-year-old, type 2 diabetes, admitted to neuro-ophthalmology clinic	Semaglutide (*N* = 169)	non-GLP-1 RAs (*N* = 234)	NAION (ICD10: H47.01 + manual reviews in source documents)	~3 years	8.9%

NAION, nonarteritic anterior ischemic optic neuropathy; SGLT2i, sodium-glucose co-transporter 2 inhibitors; non-GLP-1 RAs, nonglucagon-like peptide-1 receptor agonists; py, person-years.

Altogether, the meta-analysis was performed in three settings: A, semaglutide versus any other antidiabetic treatment (386,207 versus 626,406 patients); B, semaglutide versus non-GLP-1 RAs (279,753 versus 357,458 patients); and C, semaglutide versus SGLT2i (104,997 versus 162,269 patients). A more precise definition of the comparator groups was unavailable, and patients in those groups might have been treated with a combination of diabetes treatments (except for semaglutide for all and any other GLP-1 receptor agonist in setting B). Reported incidence rates for NAION in semaglutide groups spanned from 8.7 to 22.8 per 100,000 patient-years.

**Fig 2:**
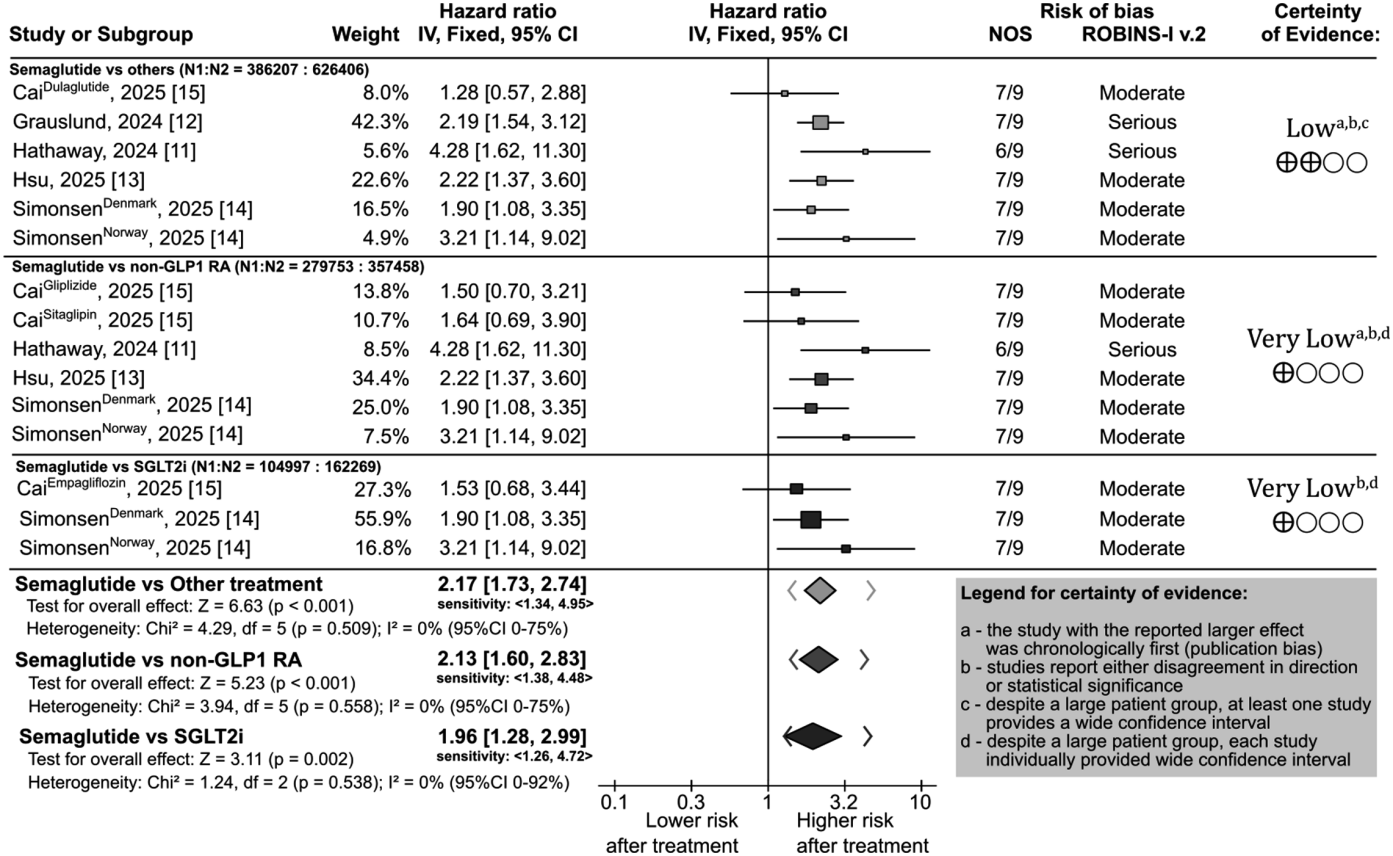
A forest plot of the included studies comparing semaglutide with other treatment regimens in patients with type 2 diabetes, along with a detailed assessment for risk of bias (Newcastle-Ottawa and ROBINS-I v.2 score) in each study, and certainty of evidence for each comparison. Details for certainty of evidence assessment: **(a)** – the study with the reported larger effect was chronologically first (publication bias); **(b)** – studies report either disagreement in direction or statistical significance; **(c)** – despite a large patient group, at least one study provides a wide confidence intervals; **(d)** – despite a large patient group, each study individually provided wide confidence intervals.

We observed a consistent significant increased hazard for NAION associated with semaglutide treatment, with HR ranging from 2.17 (95% CI [1.73, 2.74]; *p* < 0.001) for any other antidiabetic treatment, to 1.96 (95% CI [1.28, 2.99]; *p* = 0.002) for SGLT2i treatment (Fig 2), translating to the absolute risk of NAION of 0.014% (95% CI [0.005%, 0.023%]; *p* = 0.002) or 1 additional NAION case per 7,000 patients treated for 1 year.

Despite differences in study designs and definitions, statistical heterogeneity appeared to be low, with Hartung–Knapp random-effects I² of 0% (95% CI: [0%, 92%]; *p* = 0.538). However, because only five studies were included and we observed considerable uncertainty in the heterogeneity estimate, the reported low value should not be interpreted as evidence of homogeneity. The significance and direction persisted across sensitivity scenarios, with HR ranging from 1.26 to 4.95. All five studies scored 6–7 out of 9 on the NOS (Fig 2) and were rated at moderate to serious risk of bias on the ROBINS-I v.2 scale.

Funnel plots demonstrated approximate symmetry. Leave-one-out refitting indicated that the results were robust to sequentially excluding each study. Overall, these findings suggest an absence of measurable publication bias or small-study effects.

The studies covered in this synthesis were observational, at serious risk of bias, and did not fully resolve issues related to patient selection, exposure classification, and residual confounding. Although the direction of association was consistent and statistical heterogeneity was low, serious concerns about indirectness arose because NAION diagnoses were based on administrative coding without ophthalmologic confirmation, and key risk modifiers (optic disc anatomy, rate of glycemic reduction) were not accounted for.

Comparator groups were also limited, and a lack of randomization might have increased the risk of bias. Imprecision was judged to be very serious, given wide confidence intervals and a small absolute risk increase, which renders effect estimates unstable. Following the GRADE criteria, the limitations result in a low-to-very-low certainty rating, indicating that current evidence is insufficient to establish a causal relationship and that future research may substantially affect our confidence in the effect estimate (Fig 2).

### Umbrella review of systematic reviews and meta-analyses

In parallel with the primary meta-analysis, we conducted an umbrella review of higher-level evidence on the association between semaglutide and NAION. We identified 18 eligible publications, including 2 meta-analyses of RCTs [52,53], 4 meta-analyses of observational studies [75–78], 9 systematic or structured narrative reviews [43–46,48–51], and 2 statements [54,55]. We also included 4 regulatory and safety communications from the EMA and WHO [56–60]. Detailed characteristics and individual conclusions are summarized in S6–S9 Tables.

The 2 RCT meta-analyses consistently reported a low number of NAION events, with odds ratios of 1.53 (95% CI [0.53, 4.44]; *p* = 0.43) for GLP-1 RAs [53] and 3.92 (95% CI [1.02, 15.03]; *p* = 0.045) for semaglutide [52]. Both reviews emphasized that NAION is a rare event and that RCT data remain underpowered to exclude a moderate increase in relative risk. Observational meta-analyses focused mostly converged on a modestly increased relative risk of NAION. Pooled adjusted HRs generally range from about 1.35 (95% CI [1.20, 1.52]; *p* < 0.001) to 2.62 (95% CI [1.81, 3.80]; *p* < 0.001) compared with non-GLP-1 regimens [75,76,78], with one demonstrating a nonsignificant odds ratio of 1.40 (95% CI [0.97, 2.04]; *p* = 0.07) [77]. All meta-analyses cited residual confounding (by disease severity and vascular risk), heterogeneity in comparator choice (SGLT2i versus any non-GLP-1 therapy), and reliance on administrative coding of NAION. The AMSTAR-2 assessment yielded a confidence score of low to critically low, primarily due to the absence of a complete list of excluded items, the lack of a publication bias assessment, and the omission of key methodological details.

Systematic reviews discussed a repeated but heterogeneous observation signal for increased NAION risk with semaglutide, concentrated on older adults with type 2 diabetes and high vascular burden, but with very low absolute incidence and substantial uncertainty about causality. Several reviews highlighted strong disproportionality signals in FAERS and VigiBase, while emphasizing that spontaneous reporting cannot provide incidence estimates or adjust for confounding [43,47–50]. Moreover, systematic reviews highlight recurring risk factors that are potential confounders for NAION diagnosis, i.e., old age, long-standing diabetes, vascular comorbidities [44,45] (hypertension, hyperlipidemia, coronary artery disease, stroke history, obstructive sleep apnea, and smoking), and concurrent use of PDE-5 inhibitors [45]. Again, the AMSTAR-2 assessment yielded a critically low confidence score, largely due to a general lack of registered protocols, excluded-study lists, study-level bias assessments, and key method details.

The consensus statements and EMA/WHO communications were concordant in their overall interpretation [54–60]. The consensus groups did not consider the current evidence sufficient to prove a class-wide causal relationship. All statements agreed to the recommendations for heightened vigilance, particularly for patients at greater risk (e.g., prior NAION, significant optic nerve disease). Although WHO advisory committee had initially judged the evidence as inconclusive, after the EMA took more decisive action, both regulatory authorities concluded that NAION should be classified as a “very rare” (up to 1 in 10,000 users per year) side effect of semaglutide and recommended discontinuation of the drug if NAION is confirmed. Regulatory agencies and expert societies explicitly highlighted that the benefits of GLP-1 RAs outweigh potential ocular risks and should not limit access to treatment.

Taken together, the available literature provides coherent guidance, interpreting the available data as signaling a possible, but still low certainty, increase in NAION risk with semaglutide, while framing this against the drug’s substantial cardiometabolic benefits.

## Discussion

Our meta-analysis of longitudinal observational studies suggests that semaglutide use in patients with type 2 diabetes is associated with an approximately 2-fold increase in the hazard of NAION. Across comparator strata, pooled HRs ranged from 1.96 to 2.17, with broadly consistent direction and magnitude across sensitivity analyses (HRs ranging from 1.26 to 4.95) and other published meta-analyses (HRs range from 1.35 to 2.62). Our more focused approach, which restricted the intervention to semaglutide, used longitudinal designs with time-to-event analysis, and carefully handled comparators and overlapping populations, may partly explain the higher pooled HR. Importantly, systematic reviews and meta-analyses that summarize these and additional cohorts, including studies not eligible for our primary meta‑analysis [20–33,79], converge on a similar picture as evidenced by our umbrella review: a repeated, biologically plausible observational signal, but with low certainty due to residual confounding, outcome misclassification, and design limitations.

This increased hazard translates into an absolute risk difference of about 0.014% per year (95% CI [0.005%, 0.023%]; *p* = 0.002), equivalent to ~1 additional NAION case per 7,000 patients treated for 1 year—similar in magnitude to the regulatory classification of 1 extra case per 10,000 person-years of exposure. On an individual level, this represents a very small risk; nevertheless, NAION is often irreversible and can be devastating in specific risk groups (patients with monocular vision or prior NAION in the fellow eye), in which even a small excess risk matters for clinical decision-making [34–36,38–40].

However, the burden of NAION associated with semaglutide exposure should also be considered in the broader context. Recent studies estimate that ~6.2–22.7 million US adults meet the inclusion criteria for semaglutide treatment [80], with a reported real-world prescription rate of less than 15% [80]. Under these assumptions, the absolute risk difference is less than 500 excess diagnostic codes per year for NAION cases. Given the potential for overdiagnosis of ~40%–50% as reported by Cai and colleagues and others [19,81], the true NAION burden could be even lower. At the same time, the cardiometabolic benefits of semaglutide for those high-risk individuals cannot be overstated, with nearly one and a half million major adverse cardiovascular events and more than 1 million deaths which could be prevented over 10 years [82,83]. This illustrates that in patients with a substantial baseline cardiovascular risk, the expected cardiometabolic gains from semaglutide outweigh the small absolute increase in NAION risk by at least 2 orders of magnitude. Understanding both the relative and absolute dimensions, while considering both potential risks and benefits, is essential for informed clinical decision-making and clear communication with patients. An overall risk assessment should consider individual patient risk factors and the potential therapeutic benefits of semaglutide.

One of the key lines of evidence missing from our meta-analysis was post-marketing reports, which have consistently shown a marked disproportionality signal for semaglutide and NAION [29–33,79]. Both WHO VigiBase and FDA FAERS analyses show similar characteristics, with most cases occurring early after treatment initiation in older males using subcutaneous formulations. However, the reported odds ratios ranged from 11.12 (95% CI [8.15, 15.16]; *p* < 0.001) to 68.58 (95% CI [16.75, 280.67]; *p* < 0.001) [29–33,79] are unlikely to reflect the true magnitude due to the nature of spontaneous reporting. Both VigiBase and FAERS systems lack denominators and cannot provide incidence rates; they are also heavily influenced by confounding by indication, channeling bias, and differential reporting behavior once a signal attracts sufficient attention. Indeed, pharmacovigilance time-trend analyses demonstrated an increase in NAION reports during 2023–2025 [29,31,33,79]. At the same time, regulatory focus on early diabetic retinopathy worsening with GLP-1 RAs and guideline recommendations [84] for closer ophthalmic surveillance in high-risk patients or after treatment intensification might have also increased the numbers for eye screening in semaglutide recipients more than in other comparator groups, particularly in the first treatment year, which is also the median follow-up in many of the included cohorts.

This combination of enhanced surveillance, drug-specific diagnostic suspicion, and nonspecific codes could inflate NAION incidence estimates in semaglutide-treated patients. Notably, not all real-world data sets show an excess: in a large Polish private care network including over 36,000 GLP-1 RA users and matched controls with up to 2 years’ follow-up, GLP-1 RAs exposure was not an independent predictor of blindness, NAION, papilledema, or optic neuritis after multivariable adjustment, despite semaglutide being the most commonly used agent [26,85,86].

Given the limited understanding of the role of semaglutide in NAION pathogenesis, any causal link should be considered with extreme caution. The major anatomical risk factor is the small cap-to-disc ratio, also known as the “disc-at-risk,” which predisposes to pressure-flow mismatch [45]. It’s critical to remember that some NAION risk factors overlap with the semaglutide indications. Other factors, such as vascular comorbidities, male sex, and rapid changes in glucose and blood pressure (especially nocturnal dips), may predispose to NAION [44]. In this context, semaglutide’s potent glucose-lowering effect or effect on blood pressure, particularly when combined with antihypertensive or diuretic therapies, may compromise optic nerve perfusion [24,45,77,87]. Interestingly, the semaglutide NAION case series reports a more often bilateral and progressive presentation [36,37,88]. Unfortunately, no current analysis considered glucose-lowering dynamics, nor could it fully adjust for bias by indication. This sentiment is reflected in the Royal College of Ophthalmologists, the European Association for Diabetic Eye Complications, and the British Clinical Diabetologists’ statements [54,55] and mirrors evidence for the semaglutide link to diabetic retinopathy [48].

Nonetheless, the clinical message is clear. Both diabetologists and ophthalmologists should be aware of this rare but serious potential adverse event, especially in patients at higher risk—those with a previous history of NAION or significant optic nerve disease. Careful glucose titration and blood pressure monitoring, especially during the first 12 months of treatment, could be considered a safety approach [32]. Shared decision-making is essential and should involve an individualized risk-benefit assessment that carefully weighs metabolic goals against ocular safety [54,55]. For high-risk individuals, alternative glucose-lowering therapies could be considered or preferred (DDP-4i, SGLT2i, bariatric surgery) [54]. Efforts should also focus on proactive risk management of established and modifiable NAION risk factors. If NAION is confirmed, semaglutide should be stopped [56–60].

To our knowledge, this is the fifth meta-analysis evaluating the risk of NAION in patients with type 2 diabetes treated with GLP-1 RAs based on observational studies [75–78], and the second to focus specifically on semaglutide [75]. Distinctively to previous meta-analyses, our work focuses on inter-study correlation (through addressing the issue of overlapping datasets), performs a comprehensive sensitivity analysis, and provides robust pooled HR estimates. Our results are in opposition to those recently published by Özbek and colleagues [77], with the reason for the discrepancy described in detail in the S6 Table. Briefly, we suspect the discrepancy arises from differences in the design and use of published adjusted models HR rather than recalculating HR to OR. Our results are more in line with those of Goldenberg and colleagues [76] (HR of 1.60, 95% CI [1.12, 2.31]; *p* = 0.01) and Ho and colleagues (HR of 1.35, 95% CI [1.20, 1.52]; *p* < 0.001) [78]. Two other meta-analyses of RCTs, despite conflicting results, highlight the common limitation of insufficient sample size [52,53]. Indeed, to observe a doubling of the risk of a rare event such as NAION, extremely large cohorts and long follow-up are necessary [41]. Although data from RCTs are probably best suited for comprehensive detection of adverse events due to rigorous safety monitoring, they are also likely to under-represent high-risk patients.

Our meta-analysis also has many limitations. First, we focused exclusively on retrospective, mostly registry-based observational studies that reported hazard ratios. As such, publication and reporting biases could not be excluded, possibly inflating the reported measures. Source studies were susceptible to misclassifications of NAION, with only Cai and colleagues [19] and Hathaway and colleagues [15] utilizing more specific diagnostic criteria. The lack of a dedicated diagnostic code for NAION forced most studies to rely on administrative coding, which may not accurately distinguish true NAION cases. This introduces a risk of outcome misclassification that can bias hazard ratio estimates upward or downward. Additionally, the absence of detailed clinical data on key risk modifiers, such as optic disc anatomy and glycemic reduction rates, limits the ability to adjust for confounding. These methodological limitations highlight the need for cautious interpretation of results and support the importance of prospective studies with rigorous ophthalmologic confirmation of NAION diagnosis. Another limitation of our analysis stems from the broad age range of patients included in the source studies. Two studies included patients as young as 12 years [15,16], whereas others included individuals over 18; this was necessary to provide comprehensive coverage of the relevant literature. However, NAION is predominantly an age-related disorder, with incidence increasing substantially in older populations, typically above 40 years of age [41,42]. The inclusion of younger patients, who are at very low risk for NAION, likely dilutes the true hazard ratio for semaglutide in the older population, who are the primary recipients of this therapy. Thus, those studies may have underestimated the effect size. It should also be noted that treatment duration and doses were not comprehensively reported. Despite extensive statistical adjustment, residual covariance of factors not recorded in registries, such as frequency of ophthalmological consultations, smoking status, or history of cardiovascular disease, might have persisted. We were unable to resolve biases arising from potentially overlapping populations, owing to the inclusion of smaller national registries in multinational databases and the complexity of type 2 diabetes treatment regimens.

Finally, registry-based observational studies may be susceptible to various biases, particularly in the reporting of adverse events, which can lead to unwarranted public concern if not carefully scrutinized. While such studies often provide valuable data, their results should be interpreted cautiously given inherent methodological limitations. Considering available literature, the absence of evidence cannot be assumed as evidence of absence. As such, we call for the research community to conduct further large-scale, prospective, controlled studies, comprehensive pharmacoepidemiologic analyses, and target-trial emulations to confirm or refute the potential association between semaglutide and NAION. Future prospective studies with robust control groups and mechanistic studies are needed to elucidate how semaglutide may influence NAION risk, including its effects on optic nerve perfusion, microcirculation, and endothelial function. It may also be helpful to identify populations at higher risk of NAION, such as individuals with pre-existing ocular conditions or cardiovascular disease, to tailor risk-management strategies.

To conclude, our study indicates a potential association between semaglutide and NAION, though the absolute risk remains low. Given the multifactorial, incompletely elucidated pathogenesis of NAION, it seems unlikely that semaglutide is the sole contributor to its development. However, a direct triggering effect in some patients cannot be entirely excluded. Greater emphasis should be placed on identifying potential ophthalmological risk factors for NAION in patients treated with semaglutide. While clinical vigilance cannot be overstated, clinical decisions should be individualized, with particular emphasis on careful management of established NAION risk factors in patients treated with semaglutide.

### Artificial intelligence (AI)-associated technologies

We disclose that no AI-based technologies were used in the analysis or manuscript preparation.

## Supporting information

S1 Table Details on the primary included studies—design, PICO, and follow-up period. (PDF)

S2 Table Further details on the primary included studies—definition and route of semaglutide use, assessed confounding factors, and adjustment method. (PDF)

S3 Table Characteristics of additional cohort studies (design, population, exposures, comparators, outcomes, and follow-up) and reasons for exclusion from the primary analysis. (PDF)

S4 Table Non-cohort study designs and pharmacovigilance analyses (design, population, outcome definitions, follow-up, exclusion reasons, and reported effect estimates). (PDF)

S5 Table Inter-study correlation assessment through the source dataset and potential overlap—sensitivity analysis studies marked with *. (PDF)

S6 Table Summary of meta-analyses on GLP-1 RAs and NAION. (PDF)

S7 Table Narrative and mixed-methods reviews on GLP-1 RAs and NAION (context, data sources, role of NAION, and authors’ interpretation). (PDF)

S8 Table Positions of professional societies and expert groups on the semaglutide–NAION association (interpretation of evidence and practical recommendations). (PDF)

S9 Table Regulatory and global safety communications (EMA, WHO) regarding NAION as a potential or confirmed very rare adverse effect of semaglutide. (PDF)

S10 Table Funding sources and stated funder roles for each included or related study. (PDF)

S11 Table Domain-level risk of bias and overall quality scores (ROBINS-I and NOS) for observational studies. (PDF)

S1 Fig Forest plot of the meta-analysis for the non-semaglutide comparison using crude hazard ratios. (PDF)

S2 Fig Forest plot of the meta-analysis for the non-semaglutide comparison using crude hazard ratios with exclusion of overlapping cohorts. (PDF)

S3 Fig Forest plot of the meta-analysis for the non-GLP-1 RA comparison using crude hazard ratios. (PDF)

S4 Fig Forest plot of the meta-analysis for the non-GLP-1 RA comparison using crude hazard ratios with exclusion of overlapping cohorts. (PDF)

S5 Fig Forest plot of the meta-analysis for the SGLT2i comparison using crude hazard ratios. (PDF)

S6 Fig Forest plot of the meta-analysis for the non-semaglutide comparison using the more sensitive NAION definition. (PDF)

S7 Fig Forest plot of the meta-analysis for the non-semaglutide comparison using the more sensitive NAION definition with exclusion of overlapping cohorts. (PDF)

S8 Fig Forest plot of the meta-analysis for the non-GLP-1 RA comparison using the more sensitive NAION definition. (PDF)

S9 Fig Forest plot of the meta-analysis for the non-GLP-1 RA comparison using the more sensitive NAION definition with exclusion of overlapping cohorts. (PDF)

S10 Fig Forest plot of the meta-analysis for the SGLT2i comparison using the more sensitive NAION definition. (PDF)

S1 PRISMA Checklist PRISMA 2020 checklist for reporting of systematic reviews. PRISMA 2020 checklist for reporting of systematic reviews. All 27 items of the PRISMA 2020 statement were addressed; the column ‘Location where item is reported’ specifies the section and paragraph(s) in the manuscript where each item is documented. Adapted from: Page MJ, McKenzie JE, Bossuyt PM, Boutron I, Hoffmann TC, Mulrow CD, and colleagues. The PRISMA 2020 statement: an updated guideline for reporting systematic reviews. *BMJ* 2021;372:n71. https://doi.org/10.1136/bmj.n71. Licensed under CC BY 4.0 (https://creativecommons.org/licenses/by/4.0/).
(PDF)

S1 PROSPERO PROSPERO protocol registration form. (PDF)

## Abbreviations

CIs: confidence intervals
EMA: European Medicines Agency’s
FDA: Food and Drug Administration
GLP-1 RAs: glucagon-like peptide-1 receptor agonist
GRADE: Grading of Recommendations Assessment, Development and Evaluation
HRs: hazard ratios
ICD-10: International Classification of Diseases Ten
MECIR: Methodological Expectations of Cochrane Intervention Reviews
NAION: nonarteritic anterior ischemic optic neuropathy
NOS: Newcastle-Ottawa Scale
PROSPERO: Prospective Register of Systematic Reviews
RCT: randomized controlled trials
ROBINS-I: Risk of Bias in Non-Randomized Studies of Interventions
SGLT2i: sodium-glucose co-transporter 2 inhibitor
T2DM: type 2 diabetes
WHO: World Health Organization
