# Supplementary material for: Semaglutide-associated risk of nonarteritic anterior ischemic optic neuropathy in patients with type 2 diabetes: A systematic review and meta-analysis of observational studies
Source: PLoS Med. 2026 May 21;23(5):e1005064. doi: 10.1371/journal.pmed.1005064 (PMC13221145; doi:10.1371/journal.pmed.1005064)
Supplement: S1 Table — (PDF) [file pmed.1005064.s001.pdf]

Table S1. Details on the primary included studies – design, PICO, and follow-up period.

Study	Design	Population	Intervention	Comparator	Outcome definition	Follow-up
**Cai et al. 10.1001/jamaophthalmol.2024.6555**	Multi-database OHDSI/OMOP active-comparator new-user cohorts	Adults ≥18 y with T2D on metformin, starting second-line therapy	Semaglutide (subcutaneous)	(1) Dulaglutide (GLP-1 RA)<br>(2) Empagliflozin (SGLT2i)	Incident NAION defined by ICD-10 diagnosis, confirmed by a second NAION code within 80 days (sensitive/specific algorithms)	Dec 1, 2017 – Dec 31, 2023
**Grauslund et al. 10.1186/s40942-024-00620-x**	National registry-based prospective cohort (Denmark)	All adults ≥18 y with T2D (106,454 sema users / 317,698 never-sema)	≥1 prescription of once-weekly semaglutide (Ozempic®, ATC A10BJ06)	Persons with T2D without any redeemed semaglutide prescription in the period	NAION via diagnostic code H470C in Danish National Patient Registry; risk expressed as HR	Dec 1, 2018 – Dec 31, 2023
**Hathaway et al. 10.1001/jamaophthalmol.2024.2296**	Retrospective matched cohort, single academic neuro-ophthalmology registry (Mass Eye and Ear)	Patients ≥12 y referred for presumed neuro-ophthalmic indications (T2D / overweight/obesity subsets)	Prescriptions for semaglutide (for T2D or weight loss)	Non–GLP-1 RA medications for T2D or weight loss	NAION identified via ICD-10 H47.01, text search (“NAION”), and manual chart review by neuro-ophthalmology faculty	Dec 1, 2017 – Nov 30, 2023
**Hsu et al. 10.1001/jamaophthalmol.2025.0349**	Cohort study using TriNetX network	Patients with diabetes ≥12 y, no prior NAION	Semaglutide exposure (≥2 encounters coded as semaglutide use)	Non–GLP-1 RA antidiabetic medications	First NAION (ICD-10-CM H47.01x) after index; HRs at 1–4 years	Oct 1, 2019 – Dec 31, 2023
**Simonsen et al. 10.1111/dom.16316**	New-user active-comparator cohort, national registries (Denmark + Norway)	(1) Danish new users of semaglutide or SGLT2i (n=44,517 / 84,814) (2) Norwegian new users (n=16,860 / 34,153)	Semaglutide initiation	SGLT2i initiation	Country-specific ICD-10 NAION codes (H47.0C in Denmark; H47.0 in Norway); HRs estimated separately and pooled	Denmark: 2018–Jun 2024; Norway: 2018–May 2022

PICO – Population, Intervention, Comparator, Outcome; OHDSI – Observational Health Data Sciences and Informatics; OMOP – Observational Medical Outcomes Partnership; T2D – Type 2 diabetes; y – Years; GLP-1 RA / GLP 1 RA / GLP 1RAs – Glucagon-like peptide-1 receptor agonist(s); SGLT2i – Sodium–glucose cotransporter-2 inhibitor; NAION – Non-arteritic anterior ischemic optic neuropathy; ICD-10 – International Classification of Diseases, 10th Revision; ICD-10 CM – International Classification of Diseases, 10th Revision, Clinical Modification; ATC – Anatomical Therapeutic Chemical (drug classification system); HR / HRs – Hazard ratio / hazard ratios; n – Sample size (number of participants)
