# Supplementary material for: Semaglutide-associated risk of nonarteritic anterior ischemic optic neuropathy in patients with type 2 diabetes: A systematic review and meta-analysis of observational studies
Source: PLoS Med. 2026 May 21;23(5):e1005064. doi: 10.1371/journal.pmed.1005064 (PMC13221145; doi:10.1371/journal.pmed.1005064)
Supplement: S2 Table — (PDF) [file pmed.1005064.s002.pdf]

Table S2. Further details on the primary included studies – definition and route of semaglutide use, assessed confounding factors, adjustment method.

Article	Definition of semaglutide use	Route	Confounding factors	Adjustment method
**Cai et al. 10.1001/jamaophthalmol.2024.6555**	New users of second-line GLP-1 RA (semaglutide or comparators), after prior T2D diagnosis and metformin; no prior GLP-1 RA	Subcutaneous semaglutide	Age, sex, calendar time, comorbidities, diabetes severity, prior meds/procedures, health-care utilization, plus extensive covariate set aligned with OHDSI practices	Large-scale propensity-score modelling and 1:1 matching within each database; meta-analysis across databases
**Grauslund et al. 10.1186/s40942-024-00620-x**	≥1 redeemed prescription for once-weekly semaglutide in Danish National Prescription Registry	Subcutaneous	Sex, age, marital status, diabetes duration, HbA1c, eGFR, CVD history, use of insulin, statins, and antihypertensives	Multivariable Cox proportional hazards regression
**Hathaway et al. 10.1001/jamaophthalmol.2024.2296**	Prescription for semaglutide documented in single-center registry (for T2D or weight loss); matched to non–GLP-1 RA users	Primarily subcutaneous (oral form rare; not analysed separately)	Age, sex, hypertension, T2D, obstructive sleep apnea, obesity, hyperlipidemia, CAD, and absence of CKD, MEN2, thyroid tumours, pancreatitis	Propensity-score matching with Cox models for NAION
**Hsu et al. 10.1001/jamaophthalmol.2025.0349**	Semaglutide users defined by ≥2 encounters with semaglutide codes in TriNetX; no prior NAION	Route not separated (predominantly subcutaneous Ozempic/Wegovy; oral Rybelsus use relatively rare)	Race/ethnicity, sex, age, BMI, HbA1c, LDL, comorbidities (HTN, OSA, CVD, etc.), and health-care setting	TriNetX built-in propensity-score matching and survival analysis
**Simonsen et al. 10.1111/dom.16316**	New users of semaglutide vs new users of SGLT2i in Denmark/Norway (first fill in national prescription registries; ≥2 years look-back)	Route not separated (Ozempic once-weekly s.c.; oral semaglutide minimally used during study)	Age, sex, calendar year, type of prescriber (Denmark), markers of diabetes severity, systemic NAION risk factors, specific comorbidities and co-medications	Multivariable Cox models; data from the two countries analysed separately and combined

GLP-1 RA / GLP 1 RA – Glucagon-like peptide-1 receptor agonist; T2D – Type 2 diabetes; OHDSI – Observational Health Data Sciences and Informatics; HbA1c – Glycated haemoglobin A1c; eGFR – Estimated glomerular filtration rate; CVD – Cardiovascular disease; CAD – Coronary artery disease; CKD – Chronic kidney disease; MEN2 – Multiple endocrine neoplasia type 2; BMI – Body mass index; LDL – Low-density lipoprotein cholesterol; HTN – Hypertension; OSA – Obstructive sleep apnea; s.c. – Subcutaneous; PS – Propensity score; PSM – Propensity score matching
