# Supplementary material for: Semaglutide-associated risk of nonarteritic anterior ischemic optic neuropathy in patients with type 2 diabetes: A systematic review and meta-analysis of observational studies
Source: PLoS Med. 2026 May 21;23(5):e1005064. doi: 10.1371/journal.pmed.1005064 (PMC13221145; doi:10.1371/journal.pmed.1005064)
Supplement: S3 Table — (PDF) [file pmed.1005064.s003.pdf]

Table S3. Characteristics of additional cohort studies (design, population, exposures, comparators, outcomes, follow-up) and reasons for exclusion from the primary analysis.

| Study | Design | Population | Intervention | Comparator | Outcome definition | Follow-up | Exclusion reason | Reporting |
|---|---|---|---|---|---|---|---|---|
| **Chou et al. 10.1016/j.ophtha.2024.10.030, 2-11-2024** | Retrospective cohort study | Adults >18 years, T2D, obesity and other indications (TriNetX) | Semaglutide (18,657) | Non-GLP-1RA (18,657; PSM) | ICD-10 H47.01 | ~3 years | Excluded as overlapping cohort | HR 1.51 (95%CI 0.71-3.25) |
| **Fung et al. 10.1001/jamaophthalmol.2025.2299, 31-07-2025** | Retrospective cohort study | ≥65 years with T2D (US Medicare claims) | Semaglutide (188,922) | Comparable second-line antidiabetic drugs (no GLP-1RAs) | ICD-9 (377.41) and ICD-10 (H47.01) | Median ~3.7 years | Overlapping US claims population, excluded to avoid double counting | HR 1.39 (95%CI 1.13-1.72) |
| **Tesfaye et al. 10.1111/dom.70200, 17-10-2025** | New-user, active-comparator cohort study | Adults with T2D initiating GLP-1RA or SGLT2i (no prior ION; national insurance claims) | GLP-1 receptor agonists (482,912) | SGLT2 inhibitors (482,912; PSM) | ICD-10 H47.01x plus same-day ophthalmologist/optometrist visit | Median ~6.7 months | Semaglutide-specific HR is only reported as part of a meta-analysis | HR 2.78 (95%CI 1.39-5.56) |
| **Ramsey et al. 10.1001/jamanetworkopen.2025.26321, 11-08-2025** | Retrospective cohort study | Adults ≥18 years with T2D and HbA1c ≥6.5% (TriNetX) | GLP-1RAs (185,066) | No GLP-1RA (N=185,066; PSM) | ICD-10-CM H47.01 | ~2 years | Overlapping TriNetX cohort and time window; excluded to avoid double counting | HR 1.26 (95%CI 0.94-1.70) |
| **Wang et al. 10.1001/jamanetworkopen.2025.26327, 11-08-2025** | Retrospective cohort / target-trial emulation | T2D adults with no prior eye disorders (TriNetX, 1,511,637 eligible) | Semaglutide or tirzepatide (79,699) | Other antidiabetic medications (N=79,699, matched) | ICD-10-CM H47.01 | ~2-year | Overlapping TriNetX dataset and calendar period; exposure combines semaglutide and tirzepatide | HR 1.76 (95%CI 1.01-3.07) |
| **Klonoff et al. 10.1177/19322968241268050, 30-07-2024** | Retrospective real-world cohort analyses | Adults >18 years treated with weight-loss agents (Arcadia.io) | Semaglutide | Other weight-loss medications | Diagnostic codes | N/A | Overlapping US EHR/claims cohort; no clearly extractable, independent semaglutide-specific HR | HR 6.84 (95%CI 0.82-56.79) |
| **Bahit et al. 10.20452/pamw.16987, 7-04-2025** | Retrospective cohort | Adults >18 years, obesity, DM, both, or other (LuxMed private EHR network, Poland) | GLP-1RAs | Matched patients without GLP-1RAs | Composite H54, H47.01, H47.1, H46 | Jan 2018 – Jun 2024 | GLP-1RA class-level with a composite ocular outcome; no NAION-specific hazard ratios for semaglutide | HR 1.53 (95%CI 0.98-2.37) |

T2D – Type 2 diabetes; GLP-1 RA / GLP 1RA / GLP 1RAs – Glucagon-like peptide-1 receptor agonist(s); SGLT2i / SGLT2 inhibitors – Sodium–glucose cotransporter-2 inhibitor(s); PSM – Propensity score matching; ICD-9 – International Classification of Diseases, 9th Revision; ICD-10 – International Classification of Diseases, 10th Revision; HR – Hazard ratio; CI – Confidence interval; DM – Diabetes mellitus; US – United States; EHR – Electronic health record; NAION – Non-arteritic anterior ischemic optic neuropathy; ION – Ischemic optic neuropathy
