# Supplementary material for: Semaglutide-associated risk of nonarteritic anterior ischemic optic neuropathy in patients with type 2 diabetes: A systematic review and meta-analysis of observational studies
Source: PLoS Med. 2026 May 21;23(5):e1005064. doi: 10.1371/journal.pmed.1005064 (PMC13221145; doi:10.1371/journal.pmed.1005064)
Supplement: S4 Table — (PDF) [file pmed.1005064.s004.pdf]

Table S4. Non-cohort study designs and pharmacovigilance analyses (design, population, outcome definitions, follow-up, exclusion reasons, reported effect estimates).

Study	Design	Population	Intervention	Comparator	Outcome definition	Follow-up	Exclusion reason	Reporting
**Abbass et al. 10.1016/j.ajo.2025.02.025, 20-Feb-2025**	Retrospective cohort study	≥12 years with T2D (TriNetX)	Semaglutide (107,662)	Non–GLP-1RA (107,662; PSM)	ICD-10 code H47.01	1, 3, 5 years	Reported risk ratios (relative risk) at fixed time windows rather than hazard ratios	RR 0.7 (95%CI 0.523-0.937)
**Nagdeve et al. 10.1001/jamaophthalmol.2025.2332, 24-07-2025**	Case–control	Adults ≥18 years from US claims/EHR data, stratified by diabetes and obesity status	Semaglutide (1,802)	Individuals never exposed to semaglutide / GLP-1RAs	ICD-9 377.41 and ICD-10 H47.01, H47.011, H47.012, H47.013, H47.019	1, 2, ≥3 years	Odds ratios for prevalence from case–control design, not hazard ratios	OR 2.03 (95%CI 0.82-5.02)
**Azab et al. 10.1016/j.orcp.2025.01.011, 7-02-2025**	Register-based pharmacovigilance study	All FDA Adverse Event Reporting System (FAERS) reports	Semaglutide	All other drugs in FAERS	MedDRA	N/A	Only disproportionality metrics reported (PRR, ROR); no incidence rates or hazard ratios	ROR 11.36 (95%CI 8.33-15.49)
**Procacci et al. 10.1016/j.orcp.2025.03.001, 24-03-2025**	Register-based pharmacovigilance study	All FAERS reports involving semaglutide	Semaglutide	All other drugs in FAERS	MedDRA	N/A	Disproportionality-only analysis (PRR, ROR); no hazard ratios or person-time	ROR 17.57 (95%CI 13.93-21.90)
**Suresh et al. 10.1007/s10792-025-03760-7, 30-09-2025**	Retrospective post-marketing pharmacovigilance study	ICSRs in WHO VigiBase	Semaglutide-(73,636 reports)	All other drugs in VigiBase	MedDRA	N/A	No incidence rates or hazard ratios	N/A
**Cheng et al. 10.1007/s40618-025-02712-3, 29-09-2025**	Multi-database pharmacovigilance assessment	Spontaneous reports in FAERS and VigiBase	GLP-1RAs	All other drugs in FAERS/VigiBase	MedDRA	N/A	Disproportionality analysis only (ROR); no hazard ratios or person-time	ROR 40.18 (95%CI 21.05-76.7)
**Lakhani et al. 10.1016/j.ajo.2025.05.007, 16-05-2025**	Register-based pharmacovigilance study	FAERS and VigiBase reports from >180 countries	Semaglutide + tirzepatide	Other drugs (FAERS) and specific comparators (VigiBase)	MedDRA	N/A	Disproportionality study using ROR; no hazard ratios	ROR: FAERS 11.12 (95%CI 8.15-15.16) VigiBase 68.58 (95%CI 16.75-280.67)
**Zhao et al. 10.1186/s12886-025-04096-7, 28-04-2025**	Register based pharmacovigilance study	FAERS	Semaglutide	Other drugs	MedDRA	N/A	Disproportionality study using ROR; no hazard ratios	ROR 9.15 (95%CI 6.38-13.12)

T2D – Type 2 diabetes; GLP-1 RA / GLP 1RA / GLP 1RAs – Glucagon-like peptide-1 receptor agonist(s); RR – Relative risk; OR – Odds ratio; CI – Confidence interval; FAERS – Food and Drug Administration Adverse Event Reporting System; FDA – (U.S.) Food and Drug Administration; MedDRA – Medical Dictionary for Regulatory Activities; PRR – Proportional reporting ratio; ROR – Reporting odds ratio; ICSRs / ICSR – Individual case safety report(s); WHO – World Health Organization; VigiBase – WHO global database of individual case safety reports; N/A – Not applicable
