# Supplementary material for: Semaglutide-associated risk of nonarteritic anterior ischemic optic neuropathy in patients with type 2 diabetes: A systematic review and meta-analysis of observational studies
Source: PLoS Med. 2026 May 21;23(5):e1005064. doi: 10.1371/journal.pmed.1005064 (PMC13221145; doi:10.1371/journal.pmed.1005064)
Supplement: S5 Table — (PDF) [file pmed.1005064.s005.pdf]

Table S5. Inter-study correlation assessment through the source dataset and potential overlap – sensitivity analysis studies marked with *.

Source / potential overlap	Studies	Final role in analysis
National registry – Denmark	Grauslund et al. (10.1186/s40942-024-00620-x)	Included.
	Simonsen et al. (10.1111/dom.16316)	Included.
National registry – Norway	Simonsen et al. (10.1111/dom.16316)	Included.
Single-center (USA)	Hathaway et al. (10.1001/jamaophthalmol.2024.2296)	Included.
TriNetX – primarily USA	Hsu et al. (10.1001/jamaophthalmol.2025.0349)	Included.
	Abbass et al. (10.1016/j.ajo.2025.02.025)	RR, not HR; overlapping TriNetX population with Hsu.
	Chou et al. (10.1016/j.ophtha.2024.10.030)*	Earlier/smaller TriNetX analysis; overlap with Hsu.
	Ramsey et al. (10.1001/jamanetworkopen.2025.26321)*	Class-level GLP-1 RA exposure; no semaglutide-specific HR.
	Wang et al. (10.1001/jamanetworkopen.2025.26327)*	Exposure mixes semaglutide and tirzepatide.
US Medicare claims – USA	Fung et al. (10.1001/jamaophthalmol.2025.2299)*	Large ≥65 y Medicare cohort; overlaps with US claims in OHDSI/Tesfaye.
Arcadia.io – mostly USA	Klonoff et al. (10.1177/19322968241268050)*	Real-world obesity/weight-loss cohort.
Other USA	Nagdeve et al. (10.1001/jamaophthalmol.2025.2332)	Case–control with NAION prevalence and ORs; no incident HR.
OHDSI/OMOP – multinational, primarily USA	Cai et al. (10.1001/jamaophthalmol.2024.6555)	Included
	Tesfaye et al. (10.1111/dom.70200)*	Overlaps substantially with OHDSI/Medicare.
FAERS – global ICSRs	Azab et al. (10.1016/j.orcp.2025.01.011)<br>Procacci et al. (10.1016/j.orcp.2025.03.001)<br>Zhao et al. (10.1186/s12886-025-04096-7)<br>Cheng et al. (10.1007/s40618-025-02712-3)<br>Lakhani et al. (10.1016/j.ajo.2025.05.007)	No denominators; disproportionality (ROR/PRR) only.
WHO VigiBase – global ICSRs	Suresh et al. (10.1007/s10792-025-03760-7)<br>Cheng et al. (10.1007/s40618-025-02712-3)<br>Lakhani et al. (10.1016/j.ajo.2025.05.007)	No denominators; disproportionality (ROR/PRR) only.
LuxMed EHR – Poland	Bahit et al. (10.20452/pamw.16987)*	GLP-1 RA class-level, composite ocular outcome, and no semaglutide-specific NAION HR

USA / US – United States of America; EHR – Electronic health record; GLP-1 RA / GLP 1RA – Glucagon-like peptide-1 receptor agonist; OHDSI/OMOP – Observational Health Data Sciences and Informatics / Observational Medical Outcomes Partnership; FAERS – Food and Drug Administration Adverse Event Reporting System; ICSRs – Individual case safety reports; WHO VigiBase – World Health Organization global database of individual case safety reports
