# Supplementary material for: Semaglutide-associated risk of nonarteritic anterior ischemic optic neuropathy in patients with type 2 diabetes: A systematic review and meta-analysis of observational studies
Source: PLoS Med. 2026 May 21;23(5):e1005064. doi: 10.1371/journal.pmed.1005064 (PMC13221145; doi:10.1371/journal.pmed.1005064)
Supplement: S6 Table — (PDF) [file pmed.1005064.s006.pdf]

Table S6. Summary of meta-analyses on GLP1-RAs and NAION.

Review (journal)	Included study types	Population / context	Intervention (focus)	Comparator(s)	Main result	Inclusion / exclusion logic	Covariates & overlapping populations	Limitations / biases & comments
**Natividade 2025, 10.1001/jamaophthalmol.2025.2489, 14 Aug 2025**	78 semaglutide RCTs; 73,640	Adults in semaglutide phase 2/3 RCTs; mostly T2D, obesity, NAFLD, HFpEF, CKD	Semaglutide	Placebo, insulin, SGLT2i, other GLP-1 RAs, other standard therapies	NAION OR 3.92 (1.02–15.03) based on 5 trials, 8 events.	Required adverse-event reporting; MedDRA "eye disorders" plus manual search for DR/NAION; excluded non-RCTs & trials without AE reporting	No patient-level adjustment (trial-level event counts); overlap risk limited to distinct trial programs; extension studies treated as separate but still small relative to total N	Majority studies lacked end-of-study eye exam or imaging; advanced DR often excluded at baseline.
**Silverii 2025, 10.1111/dom.16076, 6 Nov 2024**	RCTs only, multi-drug GLP-1 RA class (69 trials; ~144k vs 133k patient-years)	Adults with T2D or obesity	GLP-1 RAs	Placebo or active comparators (insulin, SGLT2i, other OADs, etc.)	8 ION cases on GLP-1 RAs vs 4 on comparators; pooled OR 1.53 (0.53–4.44)	Included ≥12-month RCTs reporting full SAE listings; excluded shorter trials or those without SAE tables	No covariate adjustment (trial-level counts only).	Possible under-reporting if non-serious events were not coded as SAE. RCT selection bias.
**Chen 2025, 10.1016/j.apjo.2025.100245, 15 Sept 2025**	Mixed observational + pharmacovigilance: 10 studies (US, Denmark, Taiwan, Japan), FAERS disproportionality, and case series	Adults with T2D and/or obesity exposed to semaglutide; health-system cohorts + FAERS	Semaglutide	Non-GLP-1 RA antidiabetics, non-GLP-1 weight-loss drugs; other GLP-1 RAs in some contrasts; background FAERS database	Cohort HR meta (6 studies): pooled HR 2.62 (1.81–3.80)	Included RWE studies explicitly reporting NAION with semaglutide and a comparator.	Pooled adjusted HRs when available (PMS or adjusted). Overlapping populations were not resolved.	Residual confounding and reporting bias. Specificity of coding uncertain.
**Goldenberg 2025, 10.1111/dom.70013, 29 Jul 2025**	Observational cohorts: 8 studies, 12 cohorts	Adults with T2D and/or obesity	Semaglutide or GLP-1RA	Non-GLP-1 RA therapies; for Cai 2025, empagliflozin chosen as primary comparator; for Grauslund, non-sema users (may include other GLP-1 RAs).	Pooled adjusted HR 1.60 (1.12–2.31) for NAION with semaglutide vs control.	Included cohort studies; required adjusted effect estimates. Excluded case reports, case-series, and pharmacovigilance-only FAERS studies.	Pooled adjusted HRs (PSM, multivariable). Overlapping *patients* across national datasets still possible but probably modest.	High between-study heterogeneity, moderate to serious ROBINS-I risk of bias for most cohorts (residual confounding, immortal time), NAION misclassification risk. No clear handling of comparators.
**Ozbek 2025, 10.1016/j.ejim.2025.05.011, 21 May 2025**	Observational cohorts: 6 studies + pharmacovigilance	Adults ≥18 with T2D and/or obesity	GLP-1 RAs (primarily semaglutide)	Non-GLP-1 RA antidiabetic/weight-loss agents	Pooled OR 1.40 (0.97–2.04) T2D ORs: 1.41 (0.93–2.16)	Included observational studies with NAION outcome and GLP-1 RA vs non-GLP-1 comparators; excluded case reports, reviews.	Where available, authors used adjusted ORs / HRs; details vary by primary study; no formal handling of overlapping cohorts	High heterogeneity and varying comparators; strong confounding by indication and by comparator class. Recalculation of OR not detailed – see comment below.
**Ho 2025, 10.2337/db25-1972-LB, 20 Jun 2025**	Retrospective RWE only (7 studies)	Adults on semaglutide or GLP-1 RAs vs non-users	GLP-1 RAs (primarily semaglutide)	Non-GLP-1 RA users	Pooled HR 1.35 (1.20–1.52)	Targeted inclusion of published RWE studies with HRs for NAION in semaglutide/GLP-1 RA vs non-users.	Pooled adjusted HRs; does not detail covariates; no formal handling of overlapping cohorts.	High heterogeneity; confounding by structure, indication, comparator class.

GLP-1 RA / GLP 1 RA / GLP 1RAs – Glucagon-like peptide-1 receptor agonist(s); NAION – Non-arteritic anterior ischemic optic neuropathy; RCT / RCTs – Randomized controlled trial(s); T2D – Type 2 diabetes; NAFLD – Non-alcoholic fatty liver disease; HFpEF – Heart failure with preserved ejection fraction; CKD – Chronic kidney disease; OR – Odds ratio; ION – Ischemic optic neuropathy; SGLT2i – Sodium–glucose cotransporter-2 inhibitor; OADs – Oral antidiabetic drugs; FAERS – Food and Drug Administration Adverse Event Reporting System; HR / HRs – Hazard ratio / hazard ratios; RWE – Real-world evidence; DR – Diabetic retinopathy; AE / AEs – Adverse event(s); SAE / SAEs – Serious adverse event(s); PSM – Propensity score matching; ROBINS-I – Risk Of Bias In Non-randomized Studies of Interventions; ADA – American Diabetes Association

Detailed commentary on Özbek et al.

The results of our primary meta-analysis are in opposition to those published by Özbek et al., who reported a non-significant increase in the odds of NAION with an OR of 1.41 (95% CI 0.93-2.16). We suspect the discrepancy arises from differences in the design of the meta-analytic approach and the specific focus on semaglutide rather than on any GLP-1 RA. In our meta-analysis, we structured the comparator groups to capture homogeneous studies and avoid double-counting successfully. This resulted in a drastic reduction in heterogeneity ($I2 = 0\%$, compared with 81% in Özbek et al.).

Our decision to use published hazard ratios from adjusted models was based on the apparent relevance of NAION timing in semaglutide-treated patients with type 2 diabetes and the apparent confounding from indications for semaglutide prescription, which, in observational studies, could not be reduced in other ways. Özbek et al. obtained drastically different results by recalculating HR to OR. This is particularly evident in the studies by Grauslund et al. and Hsu et al., in which a change from HR to OR yields a non-significant result. Moreover, for Abbas et al., the OR selected by Özbek et al. refer to comparison of semaglutide against matched control at 5-year follow-up rather than any GLP1 RA against matched control – this potential mismatch is critical, as recalculating the result for the other OR results in type 2 diabetes pooled OR of 1.44, 95%CI 1.01-2.06 ($I2=77\%$, Q Cochrane $p<0.001$, random effects model) and total pooled OR of 1.44, 95%CI 1.04-1.98 ($I2=85\%$, $p<0.001$).

The selection of studies by Özbek et al. is also not fully detailed, with the arbitrary exclusion of work by Cai et al. and the simultaneous inclusion of two studies with largely overlapping populations (Hsu et al. and Chou et al.). Adjusting for overlapping populations and including Cai et al., we obtained a pooled OR for type 2 diabetes comparisons of 1.54, 95%CI 1.26-1.88 ($I2=36\%$, Q Cochrane $p=0.18$, fixed effects) and a total pooled OR of 1.80, 95%CI 1.22-2.66 ($I2=70\%$, Q Cochrane $p=0.008$, random effects). The revisions described above confirm that both analyses indicate an increased risk of NAION in patients treated with GLP-1 RA or semaglutide.
