# Supplementary material for: Semaglutide-associated risk of nonarteritic anterior ischemic optic neuropathy in patients with type 2 diabetes: A systematic review and meta-analysis of observational studies
Source: PLoS Med. 2026 May 21;23(5):e1005064. doi: 10.1371/journal.pmed.1005064 (PMC13221145; doi:10.1371/journal.pmed.1005064)
Supplement: S7 Table — (PDF) [file pmed.1005064.s007.pdf]

Table S7. Narrative and mixed-methods reviews on GLP-1 RAs and NAION (context, data sources, role of NAION, and authors' interpretation).

Review (journal, year)	Context	Intervention	Comparator(s)	Main result	Review type / data sources	NAION role in review	Authors' stance on NAION signal
**Hidalgo Ramos et al., Cureus 2025 (10.7759/cureus.89656)**	Semaglutide and NAION across clinical and pharmacovigilance data	Semaglutide (all indications, any formulation)	Non-GLP-1RA antidiabetic therapies and other GLP-1RAs in cohorts; all other drugs in FAERS/VigiBase in disproportionality analyses	Observational cohorts generally report ~2–3-fold higher NAION risk with semaglutide; pharmacovigilance databases show strong NAION disproportionality; RCT meta-analyses for GLP-1RAs show no clear excess; absolute risk is very low	Systematic review (PRISMA-style); includes cohorts, pharmacovigilance studies, case series/reports; no meta-analysis	Primary outcome and main focus	Signal considered suggestive but low-certainty; possible increased NAION risk with semaglutide in susceptible patients; causality unproven; further large, rigorous studies recommended
**Amini et al., Acta Ophthalmologica 2025 (10.1111/aos.17473)**	NAION in the GLP-1RA therapy	Primarily semaglutide; GLP-1RA as context	Non-GLP-1RA antidiabetic regimens, SGLT2 inhibitors and other GLP-1RAs in summarised cohorts/meta-analyses	Several population-based cohorts show ~2–3-fold increased NAION risk with semaglutide, particularly in the first year; RCT data are neutral and underpowered; absolute risk at individual level is very low	Targeted narrative review with structured search; includes observational cohorts, RCT meta-analysis, pharmacovigilance and case reports	Primary outcome and central theme	Signal described as consistent and biologically plausible but not definitively causal; high overlap with traditional NAION risk factors; recommendation for heightened vigilance and risk-factor control rather than broad avoidance of semaglutide
**Feldman-Billard, Diabetes & Metabolism 2025 (10.1016/j.diabet.2025.101664)**	Clinical review proposing **an ocular-risk assessment and monitoring algorithm** before and during GLP-1RA therapy	GLP-1RAs, with emphasis on semaglutide in T2D and obesity	SGLT2 inhibitors and other non-GLP-1 antidiabetics in underlying studies; other GLP-1RAs for class comparison	Synthesises data showing increased NAION hazard and strong PV signals with semaglutide alongside major cardiometabolic benefits; proposes structured baseline eye assessment and close monitoring in high-risk patients	Clinical narrative review; integrates RCTs, observational cohorts, pharmacovigilance data and pathophysiology	One of the main ocular outcomes (with diabetic retinopathy)	NAION viewed as a probable but unconfirmed risk in predisposed individuals; absolute risk small; advocates risk-stratified use of GLP-1RAs with baseline ophthalmic evaluation and cautious titration
**Nadeem et al., Canadian Journal of Diabetes 2025 (10.1016/j.jcjd.2025.07.002)**	Integrative review of diabetic retinopathy and NAION in patients treated with GLP-1RAs	GLP-1RAs as a class (including semaglutide)	Placebo/standard care in RCTs; SGLT2 inhibitors and other non-GLP-1 therapies in observational studies	For DR, evidence supports possible early worsening but unclear long-term harm; for NAION, several cohorts and PV analyses indicate a semaglutide signal but with substantial residual bias and event rarity	Narrative review with structured literature search; draws on RCTs, real-world cohorts, pharmacovigilance and basic science	NAION is a secondary but explicitly discussed outcome	NAION association considered plausible but uncertain; authors emphasise role of rapid glycaemic improvement and vascular risk; recommend early ophthalmic assessment and cautious use in high-risk individuals
**Albanese et al., Diabetology 2025 (10.3390/diabetology6100117)**	Broad review of ocular effects of GLP-1RAs (retina, optic nerve, glaucoma, AMD)	GLP-1RA class (semaglutide, liraglutide, dulaglutide, etc.)	Placebo or active comparators in RCTs; non-GLP-1RA therapies or no GLP-1RA in observational and PV data	GLP-1RAs overall show neutral or potentially beneficial ocular effects in some contexts; DR data are mixed with concern about early worsening; NAION described as a rare emerging signal mainly linked to semaglutide observational and PV findings	Broad narrative review of clinical and pre-clinical literature; no NAION-specific systematic methods	NAION appears as a subsection within optic-nerve outcomes	NAION signal judged as emerging but weak and heterogeneous; insufficient data to infer causality; recommendation for awareness and monitoring without major changes to GLP-1RA prescribing based solely on NAION
**Muzurović et al., Journal of Diabetes and Its Complications 2025 (10.1016/j.jdiacomp.2025.109148)**	Commentary on semaglutide and retinal outcomes with brief consideration of NAION	Semaglutide	Placebo/standard care in RCTs; other glucose-lowering therapies in observational DR/NAION cohorts	Argues that DR concerns are likely overstated and largely driven by rapid glycaemic change; NAION is cited as a rare complication reported in few cohorts and PV series, with conflicting findings	Short narrative commentary; selective synthesis of trial and observational data	NAION mentioned briefly as an additional safety issue	NAION evidence considered very preliminary; authors caution against over-interpreting observational HRs and do not support avoiding semaglutide solely because of NAION concerns
**Biedka et al., Family Medicine & Primary Care Review 2025 (10.5114/fmpcr.2025.150180)**	Primary-care/obesity-medicine review of semaglutide as weight-loss therapy with NAION highlighted as an emerging complication	Semaglutide (mainly for obesity, also in T2D)	Indirect comparison to other weight-loss drugs and non-semaglutide antidiabetics via cited studies	Summarises NAION epidemiology and key semaglutide-NAION cohorts/PV signals; stresses that NAION is rare but potentially devastating; emphasises high background vascular risk in typical semaglutide recipients	Non-systematic narrative review using selected cohort and PV studies	NAION is a central theme (the "challenge" in the context of obesity therapy)	Suggests semaglutide may increase NAION risk in predisposed patients; acknowledges observational confounding; advocates careful patient counselling, baseline eye history/exam in high-risk subjects and rapid referral if visual symptoms

							occur
**Liu et al., Journal of Clinical Endocrinology & Metabolism 2025 (10.1210/clinem/dgaf541)**	Endocrine perspective on GLP-1RAs and NAION, including regulatory and mechanistic context	GLP-1RAs (including semaglutide and tirzepatide)	Placebo/active comparators in RCTs; non-GLP-1RA therapies and other drugs in observational and PV work	Reviews RCT meta-analyses (very few ION events, no clear excess), large observational cohorts (HRs often ~2–7 for semaglutide vs comparators) and strong NAION/optic neuropathy signals in PV data; highlights interaction between GLP-1RAs, blood pressure, perfusion and optic nerve anatomy	Expert narrative review; integrates RCTs, observational cohorts, pharmacovigilance, mechanistic studies and regulatory communications	NAION is the primary safety focus	Concludes there is a genuine pharmacovigilance/observational signal but with low causal certainty; recommends shared decision-making, extra caution in high-risk individuals and discontinuation if NAION occurs, while noting that benefits will outweigh risks for most patients
**Singh et al., Journal of the Association of Physicians of India 2025 (10.59556/japi.72.0621)**	Editorial explaining the semaglutide–NAION issue for general physicians, with analogy to other drug-associated NAION (e.g. PDE-5 inhibitors)	Semaglutide and GLP-1RAs more broadly	Non-GLP-1RA therapies in key cohort(s); conceptual comparison to other drug classes linked to NAION	Summarises classic NAION risk factors and the main semaglutide cohort findings; places semaglutide alongside other drugs where NAION associations have been debated; emphasises limitations of observational data	Editorial/opinion piece with selective citation of cohort and NAION–drug literature	NAION is the core topic but addressed at high level	Signal presented as possible but unproven; cautions against overreaction and blanket avoidance of semaglutide; supports clinical vigilance and risk-factor management rather than major changes in prescribing in the absence of stronger evidence

NAION – Non-arteritic anterior ischemic optic neuropathy; GLP-1 RA / GLP 1RA / GLP 1RAs – Glucagon-like peptide-1 receptor agonist(s); SGLT2 inhibitors / SGLT2i – Sodium–glucose cotransporter-2 inhibitor(s); FAERS – Food and Drug Administration Adverse Event Reporting System; VigiBase – WHO global individual case safety report database; RCT / RCTs – Randomized controlled trial(s); PV – Pharmacovigilance; DR – Diabetic retinopathy; T2D – Type 2 diabetes; AMD – Age-related macular degeneration; HRs – Hazard ratios; PDE-5 inhibitors – Phosphodiesterase type-5 inhibitors; PRISMA – Preferred Reporting Items for Systematic Reviews and Meta-Analyses
