# Supplementary material for: Semaglutide-associated risk of nonarteritic anterior ischemic optic neuropathy in patients with type 2 diabetes: A systematic review and meta-analysis of observational studies
Source: PLoS Med. 2026 May 21;23(5):e1005064. doi: 10.1371/journal.pmed.1005064 (PMC13221145; doi:10.1371/journal.pmed.1005064)
Supplement: S8 Table — (PDF) [file pmed.1005064.s008.pdf]

Table S8. Positions of professional societies and expert groups on the semaglutide–NAION association (interpretation of evidence and practical recommendations).

Source	Position on NAION–semaglutide link	Risk estimate/frequency	Evidence emphasised	Main regulatory/clinical recommendations	Overall benefit–risk view
**Gregory et al., Royal College of Ophthalmologists, 10.1038/s41433-025-03718-0, 15-02-2025**	Takes a cautious but non-causal stance: acknowledges a possible NAION signal with "synthetic incretins" (semaglutide, tirzepatide) but emphasises that no definite causation has been established.	No pooled estimate; notes small absolute numbers and wide CIs in positive studies; reminds that NAION incidence in the general population is 2–10/100,000 >50 yrs.	Summarises: Hathaway JAMA Ophthalmology cohort (approx. four-fold higher NAION incidence with semaglutide vs non-GLP-1) vs RCT meta-analysis finding no significant increase in NAION with GLP-1RAs; Danish registry work both for and against an association; very large US RWE and multinational EHR analyses that do not show increased NAION after adjustment; small case series of NAION with semaglutide/tirzepatide.	Recommends informing patients of the controversy; does *not* support routine pre-treatment "disc-at-risk" screening (impractical); suggests extra caution and shared decision-making in people with prior NAION or significant optic nerve disease, where alternative glucose/weight-loss strategies (DPP-4i, SGLT2i, bariatric surgery) may be preferred.	Stresses that GLP–1–based agents have significant metabolic and cardiovascular benefits and an overall low risk profile; warns against over-reaction that might deprive patients of effective therapy while the NAION question remains unresolved.
**Carter et al., European Association for Diabetic Eye Complicationsmailing and the Association of British Clinical Diabetologists, 10.1111/dom.70160, 17-09-2025**	For DR: strong consensus that early retinopathy worsening on GLP-1RAs is primarily due to *rapid HbA1c fall*, not a direct toxic effect. For NAION specifically, there was no consensus that GLP-1RAs increase risk; current evidence is considered uncertain and limited.	No quantitative NAION risk given. NAION is treated as a potential but unproven risk signal.	Panel (58 European ophthalmologists/diabetologists/obesity specialists) reviewed structured literature on DR and NAION with GLP-1RAs, including SUSTAIN-6, post-marketing analyses, observational NAION studies, and case reports.	Key recs: (1) Benefits of GLP-1RAs outweigh potential ocular risks and should not limit access; (2) screen people with diabetes for DR within 12 months before GLP-1RA initiation, especially if long disease duration (>10 yrs), HbA1c >10% or existing DR; (3) for patients with prior NAION or sight in only one eye, explicitly discuss possible ocular risks and consider careful BP monitoring and shared decision-making; (4) call for further research and rapid dissemination of any new NAION data.	Strongly supportive of GLP-1RA use in diabetes and obesity; ocular risks should be managed by screening and communication, not by withholding therapy except in very high-risk individuals.

NAION – Non-arteritic anterior ischemic optic neuropathy; CIs – Confidence intervals; yrs – Years; RCT – Randomized controlled trial; RWE – Real-world evidence; EHR – Electronic health record; GLP-1 RA / GLP 1RAs – Glucagon-like peptide-1 receptor agonist(s); DR – Diabetic retinopathy; HbA1c – Glycated haemoglobin A1c; DPP-4i – Dipeptidyl peptidase-4 inhibitor(s); SGLT2i – Sodium–glucose cotransporter-2 inhibitor; BP – Blood pressure; SUSTAIN 6 – Cardiovascular outcomes trial of semaglutide in type 2 diabetes (SUSTAIN programme, trial 6)
