# Supplementary material for: Semaglutide-associated risk of nonarteritic anterior ischemic optic neuropathy in patients with type 2 diabetes: A systematic review and meta-analysis of observational studies
Source: PLoS Med. 2026 May 21;23(5):e1005064. doi: 10.1371/journal.pmed.1005064 (PMC13221145; doi:10.1371/journal.pmed.1005064)
Supplement: S9 Table — (PDF) [file pmed.1005064.s009.pdf]

Table S9. Regulatory and global safety communications (EMA, WHO) regarding NAION as a potential or confirmed very rare adverse effect of semaglutide

Title	Link(s)	Position on NAION–semaglutide link	Risk estimate/frequency	Evidence emphasised	Main regulatory/clinical recommendations	Overall benefit–risk view
**EMA – PRAC minutes, Jan 13–16 2025,**	https://www.ema.europa.eu/en/documents/minutes/minutes-prac-meeting-13-16-january-2025_en.pdf	Treats NAION as a signal under investigation. PRAC states the benefit–risk of Ozempic, Rybelsus, and Wegovy remains unchanged, but asks the MAH (Novo Nordisk) to provide a detailed review of all NAION cases and the literature.	None quantified at this stage.	PSUR for semaglutide; early post-marketing reports; request for a comprehensive review of NAION cases with causality assessment.	Maintain the current marketing authorisation; require the MAH to submit, within 60 days, a full review of NAION cases and to re-evaluate the need for PI/RMP changes.	Semaglutide benefit–risk clearly positive; NAION is a safety signal requiring further data.
**EMA – PRAC minutes, June 2–5 2025 & public communication 6 June 2025**	https://www.ema.europa.eu/en/documents/minutes/minutes-prac-meeting-2-5-june-2025_en.pdf; https://www.ema.europa.eu/en/news/prac-concludes-eye-condition-naion-very-rare-side-effect-semaglutide-medicines-ozempic-rybelsus-wegovy	PRAC concludes there is sufficient evidence for a causal relationship between semaglutide and NAION. NAION is classified as an undesirable effect with frequency "very rare".	Approximate two-fold increase in NAION risk vs non-users; ≈ 1 additional NAION case per 10,000 person-years of semaglutide treatment.	Integrated review of non-clinical data, RCTs, post-marketing surveillance, and "several large epidemiological studies" suggesting ~ two-fold risk; small excess signal in clinical trials.	Update SmPC/PI for Ozempic, Rybelsus, Wegovy: add NAION under adverse reactions ('very rare') and add a warning. If patients develop sudden vision loss or rapidly worsening vision, they should seek urgent medical review; if NAION is confirmed, semaglutide should be stopped.	Explicitly states the overall benefit–risk remains favourable; action is risk communication and labelling, not restriction of use.
**WHO ACSoMP meeting, 8–9 May 2025**	https://www.who.int/publications/m/item/2025-may-acsomp-recommendations	Considers NAION and semaglutide a safety signal; evidence judged inconclusive for causality at the time of the meeting.	No formal frequency; NAION is emphasised as rare and diagnostically challenging.	Danish observational cohort (>424,000 T2D pts) showing higher NAION incidence with semaglutide plus another supportive Danish study; at least two other recent studies without a clear association. Limitations: potential bias, confounding, unclear mechanism, and rarity of NAION.	Recommends: (1) keep NAION as a signal requiring further investigation; (2) revise the Risk Management Plan to include NAION as a potential risk and plan additional pharmacovigilance. Encourages WHO to align with the regulators' advice.	Acknowledges important benefits of semaglutide; calls for strengthened PV rather than restriction. Post-meeting note records EMA's later conclusion that NAION is a very rare adverse effect.
**WHO safety alert / news release, 27 June 2025**	https://www.who.int/news/item/27-06-2025-27-06-2025-semaglutide-medicines-naion; https://pubmed.ncbi.nlm.nih.gov/40840943/	Aligns with EMA: alerts to "risk of NAION associated with semaglutide"; repeats PRAC's conclusion that NAION is a very rare side effect.	"Very rare" (≤1/10,000) and "potentially affecting up to 1 in 10,000 users"; echoes EMA's estimate.	Summarises PRAC review (non-clinical, RCTs, post-marketing, literature) and notes WHO's own NAION ICSRs in VigiBase from multiple countries.	Recommends updating PI to match EMA wording; clinicians should urgently assess patients with sudden, painless vision loss; if NAION is confirmed, stop semaglutide. RMP should include NAION as a potential risk with additional PV activities.	Confirms benefit–risk remains favourable but stresses seriousness and irreversibility of NAION; justifies a global safety alert and labelling changes rather than withdrawal.

EMA – European Medicines Agency; PRAC – Pharmacovigilance Risk Assessment Committee; NAION – Non-arteritic anterior ischemic optic neuropathy; MAH – Marketing authorisation holder; PSUR – Periodic Safety Update Report; SmPC – Summary of Product Characteristics; PI – Prescribing information; RMP – Risk Management Plan; WHO – World Health Organization; ACSoMP – Advisory Committee on the Safety of Medicinal Products (WHO); PV – Pharmacovigilance; pts – Patients; T2D – Type 2 diabetes
