# Supplementary material for: Semaglutide-associated risk of nonarteritic anterior ischemic optic neuropathy in patients with type 2 diabetes: A systematic review and meta-analysis of observational studies
Source: PLoS Med. 2026 May 21;23(5):e1005064. doi: 10.1371/journal.pmed.1005064 (PMC13221145; doi:10.1371/journal.pmed.1005064)
Supplement: S10 Table — (PDF) [file pmed.1005064.s010.pdf]

Table S10. Funding sources and stated funder roles for each included or related study.

Study	Journal	Funding / support reported	Reported role of funder
**Cai 2025** [19]	JAMA Ophthalmology	Multiple NIH grants (NEI, NLM, NHLBI, NCATS, NIDDK) and VA Informatics & Computing Infrastructure	Explicit statement that funders had **no role** in design, conduct, analysis, interpretation, or publication decisions.
**Grauslund 2024** [17]	Int J Retina Vitreous	"Funding: Not applicable."	No funder involvement described.
**Hathaway 2024** [15]	JAMA Ophthalmology	Research to Prevent Blindness (RPB) unrestricted grant to institution	Explicit statement that RPB had **no role** in study design, conduct, analysis, interpretation, or publication.
**Hsu 2025** [16]	JAMA Ophthalmology	No dedicated "Funding/Support" section; only authorship roles listed.	Not specified.
**Simonsen 2025** [18]	Diabetes Obesity Metab	Independent Research Fund Denmark (project 2025-00052B)	Funder acknowledged; no specific role stated beyond general support.
**Ramsey 2025** [23]	JAMA Network Open	No funding statement identified.	Not reported.
**Wang 2025** [24]	JAMA Network Open	NIH grants (NIA, NCATS) listed for authors	Explicit statement that funders had **no role** in design, data collection, analysis, interpretation, or publication.
**Fung 2025** [21]	JAMA Ophthalmology	Intramural research of U.S. National Library of Medicine (NIH)	Explicit statement that funder had **no role** in design, conduct, analysis, interpretation, or publication decisions.
**Nagdeve 2025** [28]	JAMA Ophthalmology	No funding statement.	Not reported.
**Suresh 2025** [30]	International Ophthalmology	"Funding: The authors have not disclosed any funding."	Not applicable.
**Cheng 2025** [31]	J Endocrinol Invest	No explicit funding section; only authorship contributions and "no conflicts" statement.	Not reported.
**Abbass 2025** [27]	Am J Ophthalmology	NIH NCATS CTSA UL1TR002548; P30EY025585; Research to Prevent Blindness Challenge Grant; Cleveland Eye Bank Foundation	Explicit statement that sponsor had **no role** in design or conduct of research.
**Lakhani 2025** [32]	Am J Ophthalmology	"Dr Muni's research is supported by the Silber TARGET Fund."	Not reported.
**Chou 2025** [20]	Ophthalmology	Taichung Veterans General Hospital grant TCVGH-1136902B	Explicit statement that sponsor had no role in design, conduct, analysis or publication.
**Azab 2025** [29]	Obesity Research & Clinical Practice	"No sources of funding were used in the conduct of this study or the preparation of this article."	Not applicable.
**Procacci 2025** [79]	Obesity Research & Clinical Practice	"This research did not receive any specific grant from funding agencies in the public, commercial, or not-for-profit sectors."	Not applicable.
**Tesfaye 2025** [22]	Diabetes Obesity & Metabolism	NIDDK R01DK138036; EP supported by PCORI and FDA grants; other NIH grants for co-authors	Explicit statement that funders were not involved in design, data collection, analysis or publication.
**Zhao 2025** [33]	BMC Ophthalmology	Guangzhou Baiyun District Dean's Fund BYY23011; Guangzhou Health Science and Technology Project 20242A011032	Not specified.
**Bahit 2025** [26]	Polish Archives of Internal Medicine	"Funding: None."	Not applicable.
**Klonoff 2024** [25]	J Diabetes Sci & Technol	"The authors received no financial support for the research, authorship, and/or publication of this article."	Not applicable.

NIH – National Institutes of Health; NEI – National Eye Institute; NLM – National Library of Medicine; NHLBI – National Heart, Lung, and Blood Institute; NCATS – National Center for Advancing Translational Sciences; NIDDK – National Institute of Diabetes and Digestive and Kidney Diseases; VA – (U.S.) Department of Veterans Affairs; RPB – Research to Prevent Blindness; CTSA – Clinical and Translational Science Award; PCORI – Patient-Centered Outcomes Research Institute; FDA – (U.S.) Food and Drug Administration; TCVGH – Taichung Veterans General Hospital
