# Supplementary material for: Semaglutide-associated risk of nonarteritic anterior ischemic optic neuropathy in patients with type 2 diabetes: A systematic review and meta-analysis of observational studies
Source: PLoS Med. 2026 May 21;23(5):e1005064. doi: 10.1371/journal.pmed.1005064 (PMC13221145; doi:10.1371/journal.pmed.1005064)
Supplement: S11 Table — (PDF) [file pmed.1005064.s011.pdf]

Table S11. Domain-level risk of bias and overall quality scores (ROBINS-I and NOS) for observational studies.

Study	D1	D2	D3	D4	D5	D6	ROBINS-I v.2 score	S1	S2	S3	S4	C1	C2	E1	E2	E3	NOS score
**Grauslund 2024, 10.1186/s40942-024-00620-x**	S	L	M	M	M	M	Serious	+	+	+	+	+	?	+	+	?	7
**Simonsen 2025, 10.1111/dom.16316**	M	L	M	M	M	M	Moderate	+	+	+	+	+	?	+	+	?	7
**Hathaway 2024, jamaophthalmol.2024.2296**	S	M	S	M	M	M	Serious	–	+	+	+	+	?	+	+	–	6
**Hsu 2025, jamaophthalmol.2025.0349**	M	L	M	M	M	M	Moderate	+	+	+	+	+	?	+	+	?	7
**Abbass 2025, 10.1016/j.ajo.2025.02.025**	S	M	S	M	M	M	Serious	+	+	+	+	+	?	+	?	?	6
**Chou 2024, 10.1016/j.ophtha.2024.10.030**	S	M	M	M	M	M	Serious	+	+	+	+	+	?	+	?	?	6
**Ramsey 2025, 10.1001/jamanetworkopen.2025.26321**	M	L	M	M	M	M	Moderate	+	+	+	+	+	?	+	+	?	7
**Wang 2025, 10.1001/jamanetworkopen.2025.26327**	M	L	M	M	M	M	Moderate	+	+	+	+	+	?	+	+	?	7
**Fung 2025, 10.1001/jamaophthalmol.2025.2299**	M	L	M	M	M	M	Moderate	+	+	+	+	+	?	+	+	?	7
**Klonoff 2025, 10.1177/19322968241268050**	S	M	S	S	M	S	Serious	+	+	+	?	+	?	+	?	–	5
**Nagdeve 2025, 10.1001/jamaophthalmol.2025.2332**	S	M	S	M	M	M	Serious	+	+	+	+	+	?	+	?	?	5
**Cai 2025, 10.1001/jamaophthalmol.2024.6555**	M	L	M	M	M	M	Moderate	+	+	+	+	+	?	+	+	?	7
**Tesfaye 2025, 10.1111/dom.70200**	M	L	M	M	M	M	Moderate	+	+	+	+	+	?	+	+	?	7
**Bahit 2025, 10.20452/pamw.16987**	S	M	M	M	S	M	Serious	+	+	+	+	+	?	?	?	–	5

D1 – Bias due to confounding; D2 – Bias in classification of interventions; D3 – Bias in selection into the study/analysis; D4 – Bias due to missing data; D5 – Bias in measurement of outcomes; D6 – Bias in selection of the reported result; ROBINS-I v.2 – Risk Of Bias In Non-randomized Studies of Interventions, version 2; S1 – Representativeness of the exposed cohort; S2 – Selection of the non-exposed cohort; S3 – Ascertainment of exposure; S4 – Demonstration that outcome of interest was not present at start of study; C1 – Comparability of cohorts on the most important factor; C2 – Comparability of cohorts on any additional factor(s); E1 – Assessment of outcome (adequacy of outcome measurement); E2 – Adequacy of follow-up duration for outcomes to occur; E3 – Adequacy of follow-up of cohorts (drop-out / loss to follow-up); NOS score – Newcastle–Ottawa Scale total quality score; S (in D1–D6 columns) – Serious risk of bias; M (in D1–D6 columns) – Moderate risk of bias; L (in D1–D6 columns) – Low risk of bias; + – Criterion clearly met; – – Criterion clearly not met; ? – Criterion unclear / not adequately reported
