# Supplementary material for: Semaglutide-associated risk of nonarteritic anterior ischemic optic neuropathy in patients with type 2 diabetes: A systematic review and meta-analysis of observational studies
Source: PLoS Med. 2026 May 21;23(5):e1005064. doi: 10.1371/journal.pmed.1005064 (PMC13221145; doi:10.1371/journal.pmed.1005064)
Supplement: S2 Fig — (PDF) [file pmed.1005064.s013.pdf]

Figure S2. Non-semaglutide comparison – crude HR and no-overlap

Study or Subgroup	log[HR]	SE	Weight	Hazard ratio IV, Fixed, 95% CI	Risk of Bias A	B	C	D	E	F	G	H	I
✓ Cai [19]	0.24686	0.41325	8.2%	1.28 [0.57 , 2.88]	+	+	+	+	+	?	+	+	?
✓ Grauslund [17]	0.943906	0.149505	62.9%	2.57 [1.92 , 3.45]	+	+	+	+	+	?	+	+	?
✓ Hathaway [15]	1.453953	0.495287	5.7%	4.28 [1.62 , 11.30]	−	+	+	+	+	?	+	+	−
✓ Hsu [16]	0.797507	0.246465	23.1%	2.22 [1.37 , 3.60]	+	+	+	+	+	?	+	+	?
✕ Simonsen [18]	0.774727	0.301988	0.0%	2.17 [1.20 , 3.92]	+	+	+	+	+	?	+	+	?
✕ Simonsen [18]	1.981001	0.576263	0.0%	7.25 [2.34 , 22.43]	+	+	+	+	+	?	+	+	?
**Total**			**100.0%**	**2.42 [1.91 , 3.05]**									

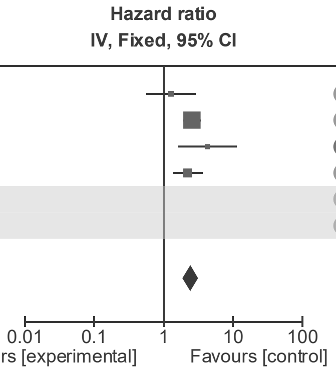

Test for overall effect: Z = 7.44 (P < 0.00001)
Test for subgroup differences: Not applicable
Heterogeneity: Chi² = 3.98, df = 3 (P = 0.26); I² = 25%

Risk of bias legend
(A) Selection 1
(B) Selection 2
(C) Selection 3
(D) Selection 4
(E) Comparability 1
(F) Comparability 2
(G) Exposure 1
(H) Exposure 2
(I) Exposure 3
