# Supplementary material for: Semaglutide-associated risk of nonarteritic anterior ischemic optic neuropathy in patients with type 2 diabetes: A systematic review and meta-analysis of observational studies
Source: PLoS Med. 2026 May 21;23(5):e1005064. doi: 10.1371/journal.pmed.1005064 (PMC13221145; doi:10.1371/journal.pmed.1005064)
Supplement: S5 Fig — (PDF) [file pmed.1005064.s016.pdf]

Figure S5. SGLT2i comparison – crude HR

Study or Subgroup	log[HR]	SE	Weight	Hazard ratio IV, Fixed, 95% CI	Risk of Bias A	B	C	D	E	F	G	H	I
✓ Cai [19]	0.81978	0.263121	50.8%	2.27 [1.36 , 3.80]	+	+	+	+	+	?	+	+	?
✓ Simonsen [18]	0.774727	0.301988	38.6%	2.17 [1.20 , 3.92]	+	+	+	+	+	?	+	+	?
✓ Simonsen [18]	1.981001	0.576263	10.6%	7.25 [2.34 , 22.43]	+	+	+	+	+	?	+	+	?
**Total**			**100.0%**	**2.52 [1.75 , 3.64]**									

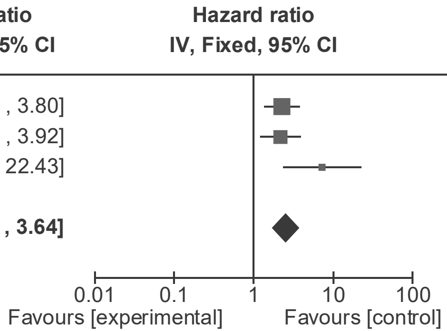

Test for overall effect: Z = 4.93 (P < 0.00001)
Test for subgroup differences: Not applicable
Heterogeneity: Chi² = 3.77, df = 2 (P = 0.15); I² = 47%

Risk of bias legend
(A) Selection 1
(B) Selection 2
(C) Selection 3
(D) Selection 4
(E) Comparability 1
(F) Comparability 2
(G) Exposure 1
