# Supplementary material for: Semaglutide-associated risk of nonarteritic anterior ischemic optic neuropathy in patients with type 2 diabetes: A systematic review and meta-analysis of observational studies
Source: PLoS Med. 2026 May 21;23(5):e1005064. doi: 10.1371/journal.pmed.1005064 (PMC13221145; doi:10.1371/journal.pmed.1005064)
Supplement: S6 Fig — (PDF) [file pmed.1005064.s017.pdf]

[illegible]
(H) Exposure 2
(I) Exposure 3

More sensitive NAION definition (where applicable)

Figure S6. Non-semaglutide comparison – sensitive NAION definition

Study or Subgroup	log[HR]	SE	Weight	Hazard ratio IV, Fixed, 95% CI	A	B	C	D	E	F	G	H	I
✓ Cai [19]	-0.072571	0.363178	10.2%	0.93 [0.46 , 1.90]	+	+	+	+	+	?	+	+	?
✓ Grauslund [17]	0.783902	0.180118	41.4%	2.19 [1.54 , 3.12]	+	+	+	+	+	?	+	+	?
✓ Hathaway [15]	1.453953	0.495287	5.5%	4.28 [1.62 , 11.30]	−	+	+	+	+	?	+	+	−
✓ Hsu [16]	0.797507	0.246465	22.1%	2.22 [1.37 , 3.60]	+	+	+	+	+	?	+	+	?
✓ Simonsen [18]	0.641854	0.288781	16.1%	1.90 [1.08 , 3.35]	+	+	+	+	+	?	+	+	?
✓ Simonsen [18]	1.166271	0.527101	4.8%	3.21 [1.14 , 9.02]	+	+	+	+	+	?	+	+	?
**Total**			**100.0%**	**2.08 [1.66 , 2.61]**									

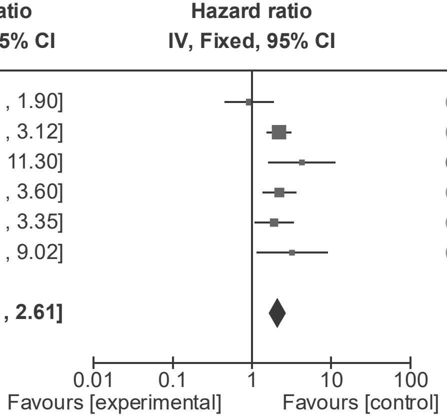

Test for overall effect: Z = 6.32 (P < 0.00001)
Test for subgroup differences: Not applicable
Heterogeneity: Chi² = 7.96, df = 5 (P = 0.16); I² = 37%

Risk of bias legend
(A) Selection 1
(B) Selection 2
(C) Selection 3
(D) Selection 4
(E) Comparability 1
(F) Comparability 2
(G) Exposure 1
(H) Exposure 2
(I) Exposure 3
