# Supplementary material for: Semaglutide-associated risk of nonarteritic anterior ischemic optic neuropathy in patients with type 2 diabetes: A systematic review and meta-analysis of observational studies
Source: PLoS Med. 2026 May 21;23(5):e1005064. doi: 10.1371/journal.pmed.1005064 (PMC13221145; doi:10.1371/journal.pmed.1005064)
Supplement: S7 Fig — (PDF) [file pmed.1005064.s018.pdf]

Figure S7. Non-semaglutide comparison - sensitive NAION definition and no-overlap

Study or Subgroup	log[HR]	SE	Weight	Hazard ratio IV, Fixed, 95% CI	Risk of Bias A	B	C	D	E	F	G	H	I
✓ Cai [19]	-0.072571	0.363178	12.9%	0.93 [0.46 , 1.90]	+	+	+	+	+	?	+	+	?
✓ Grauslund [17]	0.783902	0.180118	52.3%	2.19 [1.54 , 3.12]	+	+	+	+	+	?	+	+	?
✓ Hathaway [15]	1.453953	0.495287	6.9%	4.28 [1.62 , 11.30]	−	+	+	+	+	?	+	+	−
✓ Hsu [16]	0.797507	0.246465	27.9%	2.22 [1.37 , 3.60]	+	+	+	+	+	?	+	+	?
✗ Simonsen [18]	0.641854	0.288781	0.0%	1.90 [1.08 , 3.35]	+	+	+	+	+	?	+	+	?
✗ Simonsen [18]	1.166271	0.527101	0.0%	3.21 [1.14 , 9.02]	+	+	+	+	+	?	+	+	?
**Total**			**100.0%**	**2.06 [1.60 , 2.66]**									

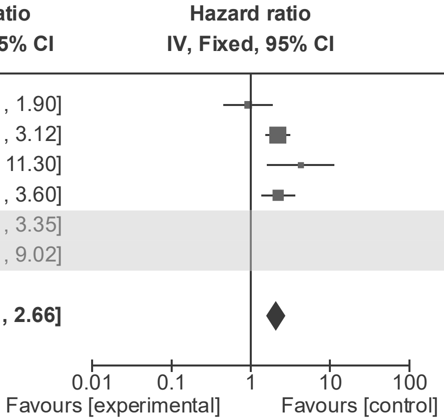

Test for overall effect: Z = 5.56 (P < 0.00001)
Test for subgroup differences: Not applicable
Heterogeneity: Chi² = 7.18, df = 3 (P = 0.07); I² = 58%

Risk of bias legend
(A) Selection 1
(B) Selection 2
(C) Selection 3
(D) Selection 4
(E) Comparability 1
(F) Comparability 2
(G) Exposure 1
(H) Exposure 2
(I) Exposure 3
