# Supplementary material for: Semaglutide-associated risk of nonarteritic anterior ischemic optic neuropathy in patients with type 2 diabetes: A systematic review and meta-analysis of observational studies
Source: PLoS Med. 2026 May 21;23(5):e1005064. doi: 10.1371/journal.pmed.1005064 (PMC13221145; doi:10.1371/journal.pmed.1005064)
Supplement: S8 Fig — (PDF) [file pmed.1005064.s019.pdf]

Figure S8. Non-GLP1 RA comparison – sensitive NAION definition

Study or Subgroup	log[HR]	SE	Weight	Hazard ratio IV, Fixed, 95% CI	A	B	C	D	E	F	G	H	I
✓ Cai [19]	0.262364	0.429028	10.5%	1.30 [0.56 , 3.01]	+	+	+	+	+	?	+	+	?
✓ Cai [19]	0.207014	0.316253	19.4%	1.23 [0.66 , 2.29]	+	+	+	+	+	?	+	+	?
✓ Hathaway [15]	1.453953	0.495287	7.9%	4.28 [1.62 , 11.30]	−	+	+	+	+	?	+	+	−
✓ Hsu [16]	0.797507	0.246465	31.9%	2.22 [1.37 , 3.60]	+	+	+	+	+	?	+	+	?
✓ Simonsen [18]	1.166271	0.527101	7.0%	3.21 [1.14 , 9.02]	+	+	+	+	+	?	+	+	?
✓ Simonsen [18]	0.641854	0.288781	23.3%	1.90 [1.08 , 3.35]	+	+	+	+	+	?	+	+	?
**Total**			**100.0%**	**1.95 [1.48 , 2.56]**									

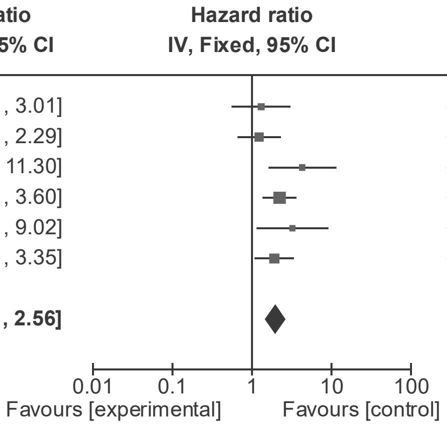

Test for overall effect: Z = 4.80 (P < 0.00001)
Test for subgroup differences: Not applicable
Heterogeneity: $Chi^2 = 6.71$, df = 5 (P = 0.24); $I^2 = 26\%$

Risk of bias legend
(A) Selection 1
(B) Selection 2
(C) Selection 3
(D) Selection 4
(E) Comparability 1
(F) Comparability 2
(G) Exposure 1
(H) Exposure 2
(I) Exposure 3
