# Supplementary material for: Semaglutide-associated risk of nonarteritic anterior ischemic optic neuropathy in patients with type 2 diabetes: A systematic review and meta-analysis of observational studies
Source: PLoS Med. 2026 May 21;23(5):e1005064. doi: 10.1371/journal.pmed.1005064 (PMC13221145; doi:10.1371/journal.pmed.1005064)
Supplement: S10 Fig — (PDF) [file pmed.1005064.s021.pdf]

Figure S10. SGLT2i comparison – sensitive NAION definition

Study or Subgroup	log[HR]	SE	Weight	Hazard ratio IV, Fixed, 95% CI	A	B	C	D	E	F	G	H	I
✓ Cai [19]	0.364643	0.314873	39.3%	1.44 [0.78 , 2.67]	+	+	+	+	+	?	+	+	?
✓ Simonsen [18]	0.641854	0.288781	46.7%	1.90 [1.08 , 3.35]	+	+	+	+	+	?	+	+	?
✓ Simonsen [18]	1.166271	0.527101	14.0%	3.21 [1.14 , 9.02]	+	+	+	+	+	?	+	+	?
**Total**			**100.0%**	**1.83 [1.25 , 2.70]**									

Hazard ratio
IV, Fixed, 95% CI

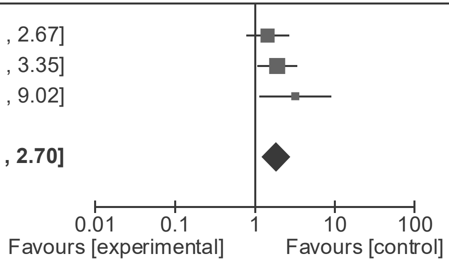

Test for overall effect: Z = 3.07 (P = 0.002)
Test for subgroup differences: Not applicable
Heterogeneity: Chi² = 1.73, df = 2 (P = 0.42); I² = 0%

Risk of bias legend
(A) Selection 1
(B) Selection 2
(C) Selection 3
(D) Selection 4
(E) Comparability 1
(F) Comparability 2
(G) Exposure 1
(H) Exposure 2
(I) Exposure 3
