# Supplementary material for: Semaglutide-associated risk of nonarteritic anterior ischemic optic neuropathy in patients with type 2 diabetes: A systematic review and meta-analysis of observational studies
Source: PLoS Med. 2026 May 21;23(5):e1005064. doi: 10.1371/journal.pmed.1005064 (PMC13221145; doi:10.1371/journal.pmed.1005064)
Supplement: S1 PROSPERO — (PDF) [file pmed.1005064.s023.pdf]

NIHR | National Institute for Health and Care Research

PROSPERO
International prospective register of systematic reviews

Semaglutide-associated risk of nonarthritic anterior ischemic optic neuropathy: a systematic review and meta-analysis of observational studies

Jędrzej Chrzanowski, Jacek Burzyński, Magdalena Walicka

Citation
Jędrzej Chrzanowski, Jacek Burzyński, Magdalena Walicka. Semaglutide-associated risk of nonarthritic anterior ischemic optic neuropathy: a systematic review and meta-analysis of observational studies. PROSPERO 2025 CRD420251025896. Available from https://www.crd.york.ac.uk/PROSPERO/view/CRD420251025896.

REVIEW TITLE AND BASIC DETAILS

Review title

Semaglutide-associated risk of nonarthritic anterior ischemic optic neuropathy: a systematic review and meta-analysis of observational studies

Condition or domain being studied

Type 2 Diabetes Mellitus; Obesity; Semaglutide; Non-arteritic Ischemic Optic Neuropathy

Population: Adults with Type 2 Diabetes and/or Obesity

Intervention: Semaglutide (or other GLP-1 Receptor Agonist)

Comparison: Non-GLP-1 Receptor Agonist Medications (no Semaglutide or GLP-1RA)

Outcome: Non-arteritic Anterior Ischemic Optic Neuropathy (NAION)

Rationale for the review

Semaglutide is increasingly prescribed for managing type 2 diabetes and obesity, and recent observational registry studies have raised concerns about a potential increased risk of Non-arteritic Anterior Ischemic Optic Neuropathy (NAION) - a rare, irreversible ocular condition that can lead to significant vision loss. Despite studies addressing this safety concern, findings have been inconsistent due to differences in study design, populations, and methodologies. This review aims to systematically synthesize observational evidence to clarify the association between semaglutide use and NAION risk and compare it with results from meta-analysis of randomized clinical trials. By pooling data from diverse national and international registries and case-control studies, and employing rigorous methods, the

review will help resolve current uncertainties, inform clinical decision-making, and potentially guide future policy and research in diabetic and obesity care.

Review objectives

The primary objective of this review is to determine whether the use of semaglutide (or other GLP1-RA) is associated with an increased risk of Non-arteritic Anterior Ischemic Optic Neuropathy (NAION) in people with type 2 diabetes and/or obesity, as evidenced by observational studies.

Secondary objectives include:

1. Assessing the magnitude of the risk (e.g., incidence rates, hazard ratios) across different populations and registries.
2. Evaluating the consistency of findings across subgroups.
3. Identifying sources of bias in registry studies and addressing their heterogeneity.

Keywords

Semaglutide; Non-Arteritic Ischemic Optic Nerve Neuropathy; Registry; Observational studies; Type 2 diabetes; Obesity

Country

Poland

ELIGIBILITY CRITERIA

Population

Included

• People aged 12 years or older,

• Diagnosed with type 2 diabetes and/or overweight and/or obesity, as defined by standard clinical criteria,

• Included in registry-based observational studies where semaglutide exposure and NAION outcomes are documented.

Excluded

• Individuals with type 1 diabetes, other types of diabetes, gestational diabetes.

• Studies lacking clear diagnostic criteria or sufficient information on semaglutide exposure.

Intervention(s) or exposure(s)

Included

Semaglutide; Glucagon Like Peptide 1 Receptor Agonists; Glycaemic-lowering agent; Weight reduction regime

• Registry-based observational studies must clearly document semaglutide exposure in people with type 2 diabetes and/or overweight and/or obesity.

• Studies should report semaglutide use as the primary exposure, for assessing its association with Non-arteritic Anterior Ischemic Optic Neuropathy (NAION).

• Studies must clearly identify a non-exposed group or a group receiving standard or usual

care, allowing for a clear contrast with semaglutide exposure.

Excluded
• Studies that do not specifically differentiate semaglutide from other GLP-1 receptor agonists or that combine semaglutide with additional interventions in a manner that obscures its individual effect

• Studies that use statistical methods inappropriate for meta-analysis, such as analyses of non-proportionality or other methods that do not allow for the acquisition of data necessary to calculate effects in meta-analysis

• Studies publish in language other than English

Comparator(s) or control(s)

Included
PICO tags selected: Weight reduction regime; Glycaemic-lowering agent; Usual Care; Placebo

- Comparators will include other antidiabetic or weight-loss therapies that do not involve semaglutide or other GLP-1 receptor agonists.

Study design

Only nonrandomized study types will be included.

Included
• non-randomized, observational studies,

• registry-based cohort studies (both prospective and retrospective),

• case–control studies,

• cross-sectional studies

• that report on semaglutide exposure and subsequent NAION outcomes.

Excluded
• interventional studies,

• narrative reviews,

• case reports and case series.

Context

The review will include registry-based observational studies conducted in various healthcare settings, such as national electronic health record systems, hospital patient registries, prescription registries, and adverse event reporting systems. Studies from high- and middle-income countries that capture routine clinical practice data will be considered, ensuring real-world evidence on semaglutide exposure and NAION outcomes. This context allows for the inclusion of diverse settings where large-scale registry data are available, reflecting the standard care and treatment practices for type 2 diabetes and obesity.

TIMELINE OF THE REVIEW

Date of first submission to PROSPERO

10 April 2025

Review timeline
Start date: 9 April 2025. End date: 8 May 2025.

Date of registration in PROSPERO
10 April 2025

AVAILABILITY OF FULL PROTOCOL

Availability of full protocol
A full protocol has been written and uploaded to PROSPERO. The protocol may be accessed through this link https://www.crd.york.ac.uk/PROSPEROFILES/ea36ff3cd6e0f55162777a930d0e980e.pdf.

SEARCHING AND SCREENING

Search for unpublished studies
Only published studies will be sought.

Main bibliographic databases that will be searched
The main databases to be searched are *PubMed* and *Scopus*.

Other important or specialist databases that will be searched
Web of Science

Search language restrictions
The review will only include studies published in English.

Search date restrictions
Databases will be searched for articles published from 1 January 2023 and before by 9 April 2025.

Other methods of identifying studies
Other studies will be identified by: *contacting authors or experts*, *looking through all the articles that cite the papers included in the review ("snowballing")*, *reference list checking* and *searching trial or study registers*.

Link to search strategy
A full search strategy is available in the full protocol as described in the *Availability of full protocol* section

Selection process
Studies will be screened independently by at least two people (or person/machine combination) with a process to resolve differences.

Other relevant information about searching and screening
Databases searched are PubMed, Scopus and Web of Science, retrieving records from January 2023 to May 2025. We included only peer-reviewed reports of original research. We will exclude reviews, meta-analyses, conference proceedings, unpublished theses, case

series, non-peer-reviewed articles. Reference lists from relevant studies was also examined to add further studies meeting the eligibility criteria.

DATA COLLECTION PROCESS

Data extraction from published articles and reports

Data will be extracted independently by at least two people (or person/machine combination) with a process to resolve differences.

Authors will be asked to provide any required data not available in published reports.

Study risk of bias or quality assessment

Risk of bias will be assessed using: *Newcastle-Ottawa*

Data will be assessed by one person (or a machine) and checked by at least one other person (or machine).

Additional information will be sought from study investigators if required information is unclear or unavailable in the study publications/reports.

Reporting bias assessment

We will assess risk of bias from missing results (reporting bias) by examining funnel plot asymmetry and performing statistical tests such as Egger's regression test. If sufficient studies are available, a trim-and-fill analysis will be conducted to estimate the impact of unpublished or missing studies on the pooled effect estimate.

Certainty assessment

We will use the Grading of Recommendations Assessment, Development, and Evaluation (GRADE) approach to assess the certainty of the evidence for each outcome. This process will involve evaluating factors such as risk of bias, inconsistency, indirectness, imprecision, and potential publication bias. Two independent reviewers will assess the evidence for each outcome using the GRADE criteria, with disagreements resolved through discussion or consultation with a third reviewer if necessary. Summary of findings tables will be created to clearly present the overall certainty of the evidence as high, moderate, low, or very low.

OUTCOMES TO BE ANALYSED

Main outcomes

The systematic review aims to identify:

1) the incidence of Non-arteritic Anterior Ischemic Optic Neuropathy (NAION) in patients exposed to semaglutide, as recorded in registry-based and case-control observational studies,

2) effect of indication (overweight or obesity, type 2 diabetes or coexistence of these conditions) on the hazard of developing NAION after exposition to semaglutide or other GLP-1RA, assessed through subgroup analyses,

3) sources of bias in registry-based observational studies reporting adverse events (RCTs).

Additional outcomes

Secondary outcomes include the magnitude of association (e.g., hazard ratio differences) across different registries and subgroups, and the identification of potential sources of bias and heterogeneity in study results.

PLANNED DATA SYNTHESIS

Strategy for data synthesis

Formal synthesis is planned. We will combine effect estimates using a random-effects meta-analysis, primarily employing the inverse variance method with the DerSimonian-Laird estimator to account for between-study heterogeneity. Missing data will be addressed by contacting study authors where feasible, and by using imputation methods when appropriate. Statistical heterogeneity will be assessed using the I^2 statistic, Cochran's Q test, and τ^2; prediction intervals will be reported if data allow. Subgroup analyses and meta-regression will be conducted to explore potential sources of heterogeneity, such as differences by population, geographic region, study design, and risk of bias. Sensitivity analyses will be performed to evaluate the robustness of the findings, including analyses excluding studies at high risk of bias. Data synthesis will be carried out using RevMan and statistical software R.

CURRENT REVIEW STAGE

Stage of the review at this submission 1 change

Review stage	Started	Completed
Pilot work	✓	✓
Formal searching/study identification	✓	✓
Screening search results against inclusion criteria	✓	✓
Data extraction or receipt of IPD	✓	✓
Risk of bias/quality assessment	✓	✓
Data synthesis	✓	✓

Review status

The review is completed.

Publication of review results

Results of the review will be published in English.

REVIEW AFFILIATION, FUNDING AND PEER REVIEW

Review team members

Dr Jędrzej Chrzanowski. ORCID: 0000-0001-6204-8622. Medical University of Lodz. Poland.

Conflict of interest

Jedrzej Chrzanowski has received consulting fees from Novo Nordisk.

Dr Jacek Burzyński. ORCID: 0000-0002-5462-8376. Medical University of Lodz. Poland.

No conflict of interest declared.

Dr Magdalena Walicka (review guarantor). ORCID: 0000-0001-8072-5279. Mossakowski Medical Research Institute, Polish Academy of Sciences; Medical Institute of the Ministry of the Interior and Administration. Poland.

No conflict of interest declared.

Named contact

Dr Jędrzej Chrzanowski (jedrzej.chrzanowski@umed.lodz.pl). ORCID: 0000-0001-6204-8622. Medical University of Lodz. Poland.

Review affiliation

Medical University of Lodz;

Mossakowski Medical Research Institute;

National Medical Institute of the Ministry of the Interior and Administration.

Funding source

Review has no specific/external funding but is supported by guarantor/review team (non-commercial) institutions.

Additional information about funding

JC is supported by Polish National Science Centre Preludium BIS 4 grant (2022/47/O/NZ5/00683), Pearls of Science (PN/01/0025/2022) and ISPAD-Breakthrough T1D Research Fellowship. The study received no explicit funding.

Peer review

There has been no peer review of this planned review.

ADDITIONAL INFORMATION

Review conflict of interest

Declared individual interests are recorded under team member details. One review team member has declared a potential conflict of interest.This review also notes the following interests:

JC has received consulting fees from Novo Nordisk. JB and MW declare no relevant conflicts of interest.

Medical Subject Headings

Adult; Anti-Obesity Agents; Diabetes Mellitus, Type 2; Glucagon-Like Peptide-1 Receptor Agonists; Humans; Incidence; Obesity; Observational Studies as Topic; Optic Neuropathy, Ischemic; semaglutide

Revision note 1 change

The manuscript has been created and is under submission.

SIMILAR REVIEWS

Check for similar records already in PROSPERO

PROSPERO identified a number of existing PROSPERO records that were similar to this one (last check made on 4 April 2025). These are shown below along with the reasons given by that the review team for the reviews being different and/or proceeding.

- Risk of Nonarteritic Anterior Ischemic Optic Neuropathy in Patients Prescribed Semaglutide: a systematic review and meta-analysis [published 5 August 2024] [CRD42024553882]. The review was acknoweldged as **similar** but the authors opted to continue because *the review will be more up to date*, *the review uses improved methods*, *there are differences in population*
- Risk of non-arteritic anterior ischaemic optic neuropathy due to semaglutide use for diabetes and weight loss: a systematic review and meta-analysis [published 16 March 2025] [CRD420250650639]. The review was acknoweldged as **similar** but the authors opted to continue because *there are differences in population*, *the review will be more up to date*, *the review uses improved methods*
- Association of semaglutide with risk of suicidal ideation: a systematic review and meta-analysis [published 13 August 2024] [CRD42024576087]. The review was judged **not to be similar**
- The Impact of Semaglutide on Cardiovascular Disease Risk: A Systematic Review and Meta-Analysis [published 12 August 2024] [CRD42024575158]. The review was judged **not to be similar**
- Safety and Efficacy of Semaglutide: An Umbrella Review [published 13 August 2024] [CRD42024576097]. The review was judged **not to be similar**
- Association of semaglutide with risk of suicidal ideation: a systematic review and meta-analysis [published 5 August 2024] [CRD42024573364]. The review was judged **not to be similar**
- Safety and Efficacy of Semaglutide: An Umbrella Review [published 16 August 2024] [CRD42024576586]. The review was judged **not to be similar**
- Association between GLP-1 agonist and risk of intestinal obstruction: a systematic review and meta-analysis [published 16 November 2024] [CRD42024610816]. The review was judged **not to be similar**
- Semaglutide and risk of coronary artery disease in patient with Type 2 Diabetes Mellitus : A Systematic Review and Meta-Analysis [published 2 December 2024] [CRD42024616539]. The review was judged **not to be similar**
- Semaglutide Use and Risk of Colorectal Cancer: A Systematic Review and Meta-Analysis [published 16 August 2024] [CRD42024576696]. The review was judged **not to be similar**
- Assessment of Macular and Peripapillary Choroidal Thickness in Nonarteritic Anterior Ischemic Optic Neuropathy by Optical Coherence Tomography [published 1 November 2022] [CRD42022369043]. The review was judged **not to be similar**
- Does Aspirin reduce the risk of non arteritic ischemic optic neuropathy in the fellow eye? A Systematic Review and Meta-analysis [published 17 December 2024] [CRD42024623286]. The review was judged **not to be similar**

- Effectiveness and Safety of Semaglutide in Weight Reduction in Patients with Overweight and Obesity: A Systematic Review with Meta-Analysis [published 5 July 2024] [CRD42024561938]. The review was judged **not to be similar**
- Cardiovascular Outcomes of Semaglutide in Patients with Chronic Kidney Disease and Type 2 Diabetes Mellitus: A systematic Review and Meta-analysis of Randomized Clinical Trials [published 29 August 2024] [CRD42024580563]. The review was judged **not to be similar**
- The Role Of Carotid Artery Disease And The Carotid Artery Hemodynamic Alterations In The Development Of Non-Arteritic Anterior Ischemic Optic Neuropathy And Non-Arteritic Posterior Ischemic Optic Neuropathy [published 3 February 2024] [CRD42024505580]. The review was judged **not to be similar**
- Association between Glp-1 Receptor Agonists and Suicidal Ideation in Type 2 Diabetes and Obesity: A Meta-Analysis [published 22 October 2024] [CRD42024603691]. The review was judged **not to be similar**
- Weight Loss Outcomes Associated With Semaglutide Treatment: A Systematic Review and Meta-Analysis [published 6 August 2024] [CRD42024573475]. The review was judged **not to be similar**
- Comparative Efficacy and Safety of Semaglutide vs. Caloric Restriction through Intermittent Fasting for Weight Management in Obese Adults Aged 40 and Older with Diabeties Type 2: A Systematic Review and Meta-Analysis [published 19 October 2024] [CRD42024598971]. The review was judged **not to be similar**
- Efficacy of Treatment for NAION: A Systematic Review and Network Meta-Analysis [published 17 April 2024] [CRD42024532781]. The review was judged **not to be similar**
- Effects of Ozempic (Semaglutide) on Diabetic Retinopathy in Type 2 Diabetes Patients [published 5 February 2025] [CRD42025641990]. The review was judged **not to be similar**

PROSPERO version history 1 change

- Version 1.1, published 02 Jul 2025
- Version 1.0, published 10 Apr 2025

Disclaimer

The content of this record displays the information provided by the review team. PROSPERO does not peer review registration records or endorse their content.

PROSPERO accepts and posts the information provided in good faith; responsibility for record content rests with the review team. The guarantor for this record has affirmed that the information provided is truthful and that they understand that deliberate provision of inaccurate information may be construed as scientific misconduct.

PROSPERO does not accept any liability for the content provided in this record or for its use. Readers use the information provided in this record at their own risk.

Any enquiries about the record should be referred to the named review contact

Prospero Protocol (1.0):

1. **Review Title (50 words max)**

Semaglutide-associated Risk of Non-arteritic Anterior Ischemic Optic Neuropathy: a Systematic Review and Meta-Analysis of Observational Studies

2. **Original Language Title**

Semaglutide-associated Risk of Non-arteritic Anterior Ischemic Optic Neuropathy: a Systematic Review and Meta-Analysis of Observational Studies

3. **Anticipated or actual start date**

9 April 2025

4. **Anticipated completion date**

8 May 2025

5. **Stage of review at time of this submission**

**Stage**	**Started**	**Completed**
Preliminary searches	Yes	Yes
Piloting of the study selection process	Yes	Yes
Formal screening of search results against eligibility criteria	Yes	Yes
Data extraction	Yes	No
Risk of bias (quality) assessment	Yes	No
Data analysis	Yes	No

6. **Review team members and their organisational affiliations**

Jędrzej Chrzanowski, MD, Department of Biostatistics and Translational Medicine, Medical University of Lodz, Poland

Jacek Burzyński, MD, Department of Biostatistics and Translational Medicine, Medical University of Lodz, Poland

Magdalena Walicka, MD, PhD, Department of Human Epigenetics, Mossakowski Medical Research Institute, Polish Academy of Sciences, Department of Internal Diseases, Endocrinology and Diabetology, National Medical Institute of the Ministry of the Interior and Administration

7. **Funding sources/sponsors.**

JC is supported by Polish National Science Centre Preludium BIS 4 grant (2022/47/O/NZ5/00683), Pearls of Science (PN/01/0025/2022) and ISPAD-Breakthrough T1D Research Fellowship. The study received no explicit funding.

8. Conflicts of interest

Jędrzej Chrzanowski has received consulting fees from Novo Nordisk.

9. Collaborators. Name and affiliation of any individuals or organizations working on review but who are not listed as review team members

N/A

10. Review question: State the review question(s) clearly and precisely. It may be appropriate to break very broad questions down into a series of related more specific questions. Questions may be framed or refined using PI(E)COS or similar where relevant.

Q1) Is the use of semaglutide associated with an increased risk of Non-arteritic Anterior Ischemic Optic Neuropathy (NAION) in adults with type 2 diabetes, as evidenced by registry-based observational studies?

Q2) Is the use of semaglutide associated with an increased risk of Non-arteritic Anterior Ischemic Optic Neuropathy (NAION) in adults with overweight or obesity, as evidenced by registry-based observational studies?

Q3) Is the use of semaglutide associated with an increased risk of Non-arteritic Anterior Ischemic Optic Neuropathy (NAION) in adults with type 2 diabetes and overweight or obesity, as evidenced by registry-based observational studies?

Q4) Are there changes in risk across different populations, registries and subgroups?

Q5) Are there apparent sources of bias in the covered registry studies?

11. Searches:

Databases searched are PubMed, Scopus and Web of Science, retrieving records from January 2023 to May 2025. We included only peer-reviewed reports of original research. We will exclude reviews, meta-analyses, conference proceedings, unpublished theses, case series, non-peer-reviewed articles. Reference lists from relevant studies was also examined to add further studies meeting the eligibility criteria.

12. Search strategy.

PubMed: (SEMAGLUTIDE OR "GLUCAGON-LIKE PEPTIDE 1"[MH] OR GLUCAGON-LIKE PEPTIDE 1 OR GLUCAGON-LIKE PEPTIDE-1 RECEPTOR OR GLUCAGON-LIKE PEPTIDES) AND (NONARTERITIC ISCHEMIC OPTIC NEUROPATHY OR

"OPTIC NEUROPATHY, ISCHEMIC"[MH] OR NAION) 2023/01/01:2025/04/09 [DP]

Scopus: (TITLE-ABS-KEY ("semaglutide") OR TITLE-ABS-KEY ("glucagon-like peptide 1") OR TITLE-ABS-KEY ("glucagon-like peptide-1 receptor") OR TITLE-ABS-KEY ("glucagon-like peptides")) AND (TITLE-ABS-KEY ("nonarteritic ischemic optic neuropathy") OR TITLE-ABS-KEY ("ischemic optic neuropathy") OR TITLE-ABS-KEY ("NAION")) AND (PUBYEAR > 2022 AND PUBYEAR < 2026)

Web of Science: TS=("semaglutide" OR "glucagon-like peptide 1" OR "glucagon-like peptide-1 receptor" OR "glucagon-like peptides") AND TS=("nonarteritic ischemic optic neuropathy" OR "ischemic optic neuropathy" OR "NAION") AND PY=(2023-2025)

13. Condition or domain being studied.

Nonarteritic anterior ischemic optic neuropathy (NAION) is a condition that results from inadequate blood flow to the optic nerve head, leading to sudden, painless vision loss. It is frequently observed in adults with systemic risk factors such as type 2 diabetes, hypertension, and obesity.

This review focuses on assessing the potential increased risk of NAION among patients receiving semaglutide - a glucagon-like peptide-1 receptor agonist used to manage type 2 diabetes and obesity based on evidence from registry-based observational studies.

14. Participants/population.

Inclusion Criteria:

- People aged 12 years or older,
- Diagnosed with type 2 diabetes and/or overweight and/or obesity, as defined by standard clinical criteria,
- Included in registry-based observational studies where semaglutide exposure and NAION outcomes are documented.

Exclusion Criteria:

- Individuals with type 1 diabetes, other types of diabetes, gestational diabetes.
- Studies lacking clear diagnostic criteria or sufficient information on semaglutide exposure.

15. Intervention(s), exposure(s).

Inclusion Criteria:

- Registry-based observational studies must clearly document semaglutide exposure in people with type 2 diabetes and/or overweight and/or obesity.
- Studies should report semaglutide use as the primary exposure, for assessing its association with Non-arteritic Anterior Ischemic Optic Neuropathy (NAION).

- Studies must clearly identify a non-exposed group or a group receiving standard or usual care, allowing for a clear contrast with semaglutide exposure.

Exclusion Criteria:

- Studies that do not specifically differentiate semaglutide from other GLP-1 receptor agonists or that combine semaglutide with additional interventions in a manner that obscures its individual effect
- Studies that use statistical methods inappropriate for meta-analysis, such as analyses of non-proportionality or other methods that do not allow for the acquisition of data necessary to calculate effects in meta-analysis
- Studies publish in language other than English

16. Comparator(s)/control.

Comparators will include other antidiabetic or weight-loss therapies that do not involve semaglutide or other GLP-1 receptor agonists.

17. Types of study to be included.

Inclusion Criteria:

- non-randomized, observational studies,
- registry-based cohort studies (both prospective and retrospective),
- case–control studies,
- cross-sectional studies
- that report on semaglutide exposure and subsequent NAION outcomes.

Exclusion criteria:

- interventional studies,
- narrative reviews,
- case reports and case series.

18. Context. Give summary details of the setting or other relevant characteristics, which help define the inclusion or exclusion criteria

The review will include registry-based observational studies conducted in various healthcare settings, such as national electronic health record systems, hospital patient registries, prescription registries, and adverse event reporting systems. Studies from high- and middle-income countries that capture routine clinical practice data will be considered, ensuring real-world evidence on semaglutide exposure and NAION outcomes. This context allows for the inclusion of diverse settings where large-scale registry data are available, reflecting the standard care and treatment practices for type 2 diabetes and obesity.

19. * Main outcome(s).

The systematic review aims to identify:

1) the incidence of Non-arteritic Anterior Ischemic Optic Neuropathy (NAION) in patients exposed to semaglutide, as recorded in registry-based and case-control observational studies,

2) effect of indication (overweight or obesity, type 2 diabetes or coexistence of these conditions) on the hazard of developing NAION after exposition to semaglutide or other GLP-1RA, assessed through subgroup analyses,

3) sources of bias in registry-based observational studies reporting adverse events (RCTs).

20. Additional outcome(s).

Secondary outcomes include the magnitude of association (e.g., hazard ratio differences) across different registries and subgroups, and the identification of potential sources of bias and heterogeneity in study results.

21. * Data extraction (selection and coding).

Step 1) Title and Abstract will be assessed for inclusion by two independent reviewers (JC, MW). In cases of doubt or lack of consensus, abstracts will be included for review.

Step 2) Full-text articles will be assessed for inclusion by two reviewers (JC, MW). Disagreements will be resolved by discussion between reviewers with arbiter (JB).

All reviewers will be involved in the data extraction process. One reviewer will extract data from each study, with at least 20% independently verified by a second reviewer to ensure agreement. The following will be extracted and recorded on a standardized data extraction sheet:

a. sample characteristics (sample size, age, sex, diagnosis, comorbidities)
b. outcome definitions and measures (NAION diagnostic criteria, follow-up duration)
c. study design features,
d. relationships between clinical covariates and events,
e. any further information on limitations,
f. funding source and possible conflicts of interest
g. quality/risk of bias assessment conducted by the primary reviewer and verified by a second reviewer.

22. * Risk of bias (quality) assessment.

The quality of included studies will be assessed using the Newcastle-Ottawa Scale, a validated tool for observational studies. This assessment will evaluate three key domains: selection of participants, comparability of study groups, and ascertainment of outcomes. At least 20% of risk of bias assessments will be conducted independently by a second reviewer to ensure reliability, with discrepancies resolved through consensus.

23. * Strategy for data synthesis.

We will provide a qualitative summary of all included studies. Where appropriate, meta-analyses will be conducted using a random-effects model to pool effect estimates for

NAION risk associated with semaglutide exposure. Heterogeneity will be assessed using I² statistics and Cochran's Q test. Subgroup and sensitivity analyses will be performed to explore sources of heterogeneity. Data synthesis will be performed using RevMan and statistical software R with appropriate libraries.

24. * Analysis of subgroups or subsets.

Subgroup analyses will be performed using RevMan and R to assess differences in treatment effects across subgroups. Exploratory subgroup analyses may be conducted if unexpected patterns emerge during data synthesis; however, these results will be clearly labeled as exploratory and interpreted with caution to avoid overgeneralization. Data analysis will be carried out using RevMan and R. Findings from subgroup analyses will be reported in the context of overall results to provide a balanced interpretation.

25. * Type and method of review.

The systematic review will follow a quantitative synthesis of observational studies examining the risk of Non-arteritic Anterior Ischemic Optic Neuropathy (NAION) associated with semaglutide exposure. It will follow Evidence-Based Medicine principles and aim to provide a comprehensive synthesis of available evidence.

The review will include:

- formulation of a research question using PICOS criteria.
- literature search across multiple databases
- clear inclusion and exclusion criteria.

quality assessment using appropriate tools.

Data synthesis via meta-analysis with a random-effects model, including subgroup analyses and heterogeneity assessment.

26. Other registration details.

N/A

27. Reference and/or URL for published protocol.

N/A

28. Dissemination plans.

We intend to publish the final systematic review and meta-analysis in a peer-reviewed journal specializing in endocrinology, ophthalmology, or pharmacovigilance. Findings will also be presented at relevant international conferences and disseminated through academic networks.

29. Keywords.

semaglutide; non-arteritic anterior ischemic optic neuropathy; observational studies; type 2 diabetes; obesity

30. Details of any existing review of the same topic by the same authors.

N/A

31. * Current review status.

Ongoing

32. Any additional information

None
